# Comparison of Argentinean microbiota with other geographical populations reveals different taxonomic and functional signatures associated with obesity

**DOI:** 10.1038/s41598-021-87365-x

**Published:** 2021-04-08

**Authors:** Susana A. Pesoa, Nestor Portela, Eduardo Fernández, Osvaldo Elbarcha, Martin Gotteland, Fabien Magne

**Affiliations:** 1 Department of Molecular Diagnosis, LACE Laboratories, Avda Velez Sarsfield 528, Córdoba, Argentina; 2 Department of Nutrition, Faculty of Medicine, University of Chile, Santiago, Chile; 3 Institute of Nutrition and Food Technology (INTA), University of Chile, Santiago, Chile; 4 Microbiology and Mycology Program, Biomedical Sciences Institute (ICBM), School of Medicine, University of Chile, Av. Independencia 1027, Independencia, Santiago, Chile

**Keywords:** Microbial communities, Metabolic disorders, Nutrition disorders

## Abstract

Accumulating evidence suggests that various genetic and environmental factors contribute to the development of obesity. Among the latter, the gut microbiota has emerged as a critical player in the regulation of human metabolism and health and the development of non-communicable chronic diseases. Considering that no information on this matter is available in Argentina, our aim was to identify the microorganisms associated with obesity as well as their potential functionality. Using high throughput sequencing of 16SrRNA bacterial gene and diverse bioinformatics tools, we observed that the gut microbiota of obese and overweight individuals differs qualitatively and quantitatively from that from their lean counterparts. The comparison of the gut microbiota composition in obese subjects from Argentina, US and UK showed that the beta diversity significantly differs among the three countries, indicating that obesity-associated microbiota composition changes according to the geographical origin of the individuals. Moreover, four distinct microbiotypes were identified in obese individuals, whose prevalence and metabolic pathway signature differed according to the country, indicating that obesity associated dysbiosis would comprise several structures. In summary, identification of distinct taxonomic signatures associated with obesity might be a novel promising tool to stratify patients based on their microbiome configuration to design strategies for managing obesity.

## Introduction

Overweight and obesity have reached epidemic proportions worldwide, currently affecting more than 600 million adults and 100 million children^1,2^. These conditions predispose to the development of severe complications such as type 2 diabetes, cardiovascular diseases, and non-alcoholic fatty liver diseases, ultimately leading to the degradation of quality of life and life expectancy^3,4^. Obesity results from complex interactions between genetic and environmental factors including dietary factors, physical activity, and modern lifestyle. More recently, gut microbiota has also been involved in the development of obesity and other noncommunicable diseases in humans^4,5^. This dynamic ecosystem, mainly inhabiting the colon, is considered a key player in the regulation of energy metabolism and inflammatory processes in the host. Changes in its composition and diversity, called dysbiosis, have been associated with obesity^6–8^. Microbiota alterations are important considering that the gut microbiome presents a vast repertoire of genes coding for enzymes involved in metabolic pathways which can be considered as complementary to those expressed by the host, performing functions essential for human physiology.

The involvement of gut dysbiosis in energy metabolism disturbances was described for the first time in germ-free mice. In their seminal study, Turnbaugh et al. reported that these animals, when transplanted with the fecal microbiota of obese mice, gained twice as much weight as those receiving the microbiota from lean donors. Metagenomic and biochemical studies showed that the microbiota of obese individuals had increased capacity to extract energy from foods^9^. In addition, the microbiota was shown to inhibit the intestinal expression of Fasting-Induced Adiposity Factor (FIAF), a hormonal factor acting as an inhibitor of lipoprotein lipase (LPL) in adipose tissue and promote adipocyte fat storage. Other mechanisms involve changes in the profile of secondary bile salts and the release, by enteroendocrine cells, of digestive hormones with anorexigenic and incretin effects^7,10–14^. On the other hand, obesity is also associated with alterations of the gut barrier function, which favor the translocation of proinflammatory bacterial components such as lipopolysaccharide (LPS) and flagellin, resulting in the so-called metabolic endotoxemia. Increased plasma LPS activates the innate immune system through Toll-like receptor 4 (TLR4) in white adipose tissue, amplifying the inflammatory tone and the subsequent development of metabolic complications^15,16^. Although a consensus has emerged on the involvement of the microbiota in obesity development, there is not yet a clear picture of the bacterial taxa involved in this condition.

While the microbiota of healthy subjects has been shown to differ according the country, it is not clear whether the same occurs for the microbiota of obese subjects^17^. Studies evaluating the association between gut microbiota and obesity have mainly focused on people living in industrialized countries of the northern hemisphere, while data from Latin America are scarce. In particular, the role of ethnicity/ancestry, lifestyle and genetic factors in the gut microbiota-obesity relationship is not yet clearly established.

In this context, the aim of this study was to describe changes in the gut microbiota composition of lean, overweight and obese Argentinean subjects, and to compare the results with those from people living in other countries to better understand how gut microbiota contributes to obesity.

## Materials and methods

### Subjects

The study was approved by the Institutional Review Board of the Romagosa-Oulton Medical Centers (Córdoba, Argentina). All the participants had to give a written informed consent prior their enrollment. Research was performed in accordance with relevant guidelines and regulations. Seventy-three volunteers were recruited in this study, who fulfilled the following inclusion criteria: BMI > 18.5 kg/m^2^ according to WHO criteria, age > 18 years, without diagnostic of gastrointestinal disease, medical treatment including intake of antibiotics, drugs or supplements that could affect gut microbiota in the last 3 months. Individuals participating in weight reduction programs and those with neuropsychological disorders, immunosuppression, tumoral and autoimmune diseases were excluded from the study.

### Fecal sample collection

A sterile and hermetic collection kit was provided to the volunteers for the collection of a fresh stool at home. The stools were frozen, brought to the laboratory within 24 h of collection, and stored at − 40 °C until analysis.

### DNA extraction

Stool samples were handled under a laminar flow hood using sterile technique. Microbial DNA was isolated from 220 mg of stool using the QIAmp DNA Stool Mini Kit (Qiagen, Germantown, MD) following the manufacturer’s standard protocol. DNA concentrations were measured using fluorometric quantitation with a Qubit 2 and the Qubit dsDNA high-sensitivity kit (Thermo Fisher Scientific Carlsbad, CA, USA) and the extracted DNA was stored at − 80 °C.

### 16S rRNA gene amplicon library preparation sequencing and taxonomic identification of bacteria

Sequencing was performed using Ion 16S Metagenomics Kit (Thermo Fisher Scientific, Carlsbad, CA, USA) on the Ion Torrent Personal Genome Machine (PGM) platform (Thermo Fisher Scientific Carlsbad, CA, USA). Briefly, libraries were generated from 20 ng of fecal DNA with the Ion 16S Metagenomics Kit, using a combination of two pools of primers targeting the V2, V4 and V8 hypervariable regions of 16S rRNA gene with the pool 1, and the V3, V6-7 and V9 regions with the pool 2. Primers were partially digested and bar-codes were ligated to the amplicons, purified using the Agencourt AMPure XP beads (Beckman Coulter; Pasadena, CA, USA) according to the manufacturer’s protocol, and stored at − 20 °C. The concentration of each 16S library was determined by qPCR using the Ion Universal Library Quantitation Kit (Thermo Fisher Scientific Carlsbad, CA, USA). The library was diluted to ~ 10 pM before template preparation. Template preparation of the barcoded libraries was performed using the Ion PGM Hi-Q View OT2 kit (Thermo Fisher Scientific Carlsbad, CA, USA) and the Ion OneTouch 2 System (Thermo Fisher Scientific Carlsbad, CA, USA). Mock community dataset was generated from a mixed bacterial genomic DNA from ATCC strains including *Escherichia coli* ATCC 25922, *Staphylococcus aureus* ATCC 25923, *Pseudomonas aeruginosa* ATCC 27853, *Enterococcus faecalis* ATCC 29212 and *Streptococcus group B*, the latter isolated and typified in LACE Laboratory. A maximum of 9 barcoded 16S samples was sequenced on a Ion 316v2 chip using the Ion PGM Hi-Q view Sequencing Kit (Thermo Fisher Scientific; Carlsbad, CA, USA) according to the manufacturer’s instructions. Automated analysis, annotation, and taxonomical assignment were generated by Qiime tools Metagenomics 16S w1.1 version 5.10 workflow in Ion Reporter SW. Curated Greengenes version 13.5 and MicroSEQ(R) 16S Reference Library v2013.1 database were used.

### United States (US) and United Kingdom (UK) datasets

Sequences of the hypervariable region V4 of the 16S rRNA gene from studies carried out in the United States^18^ and United Kingdom^19^ for which the data relative to the nutritional status of the subjects (MBI) were available.

### Comparison of different populations

From the whole PGM sequencing reads obtained in this study, only those targeting the V4 region were selected and used to compare the gut microbiota composition among the three countries. Briefly, an amplicon sequence variant (ASV) Table was built for each selected study using the DADA2 pipeline in R environment (https://benjjneb.github.io/dada2/tutorial.html). DADA2 performs quality trimming and filtering (truncQ = 2, maxN = 0, maxEE = 2), dereplication of sequences, learns the error rates and removes sequences containing potential/probable errors using default settings (denoising). The sequences from the different ASV Tables were extracted, aligned and trimmed at the same length using the mother pipeline (using the align.seqs, filter.seqs and pcr.seqs commands). Next, ASV Tables including the trimmed sequences were merged into a full-study sequence Table. Chimeras were identified and removed, and finally taxonomy was assigned. The full-study ASV Table was imported into phyloseq v.1.28.0 R Package, for further analysis^20^.

### “*Microbiotype*” classification

The probabilistic modelling of exclusive microbial metagenomics data was done by clustering the microbiome communities into exclusive metacommunities, with the Dirichlet Multinomial Mixtures Model (DMM) in the R Platform^21^. The DMM approach describes each community by a vector of taxa probabilities. These vectors are generated from the optimal number of Dirichlet mixture components selected using the minimum Bayesian Information Criterion approximation (BIC). The mixture components cluster the microbial communities into distinct groups of samples with similar composition, named from now on “microbiotype” in our study. The distinct populations were stratified according to the obesity status and geographical factor. Chi-square test was performed to compare the microbiotype frequencies between the different populations. Subsequently, the microbiotypes were imported in the phyloseq v1.28.0 R package and the abundances of the main bacterial families were determined.

### Statistical data analysis

Statistical analysis were carried out and visualized using the R version 3.6.1 software^22^. The Argentinean subjects were stratified according to their obesity status. The geographical factor was also considered for the comparison between the different populations. Alpha-diversity (Observed ASVs and Shannon index) and Beta-diversity (Bray–Curtis distances) were calculated based on the ASV Table representing the relative abundances of bacterial taxa from the microbiome v1.6.0 R package^23^. The association between BMI status and the overall microbiota composition was tested using Adonis test through the Adonis function in vegan v2.5.6 R package^24^. Differential abundance analysis was performed using the Kruskal–Wallis tests at phylum, class, order, family, genus, and species levels. False discovery rate (FDR) control based on the Benjamin-Hochberg procedure was used to correct for multiple testing. Correlation between bacterial taxa abundances and BMI values was calculated using the function “associate” in the microbiome v1.6.0 R package^23^. All data were highlighted on boxplot or heatmap using the ggplot2 v3.2.1^25^ and Complex Heatmap v2.0.0 R package^26^.

### PICRUSt analysis

PICRUSt approach was used to evaluate the functional potential of the different microbiotypes^27^. The full-study ASV Table was converted to BIOM format using the biomformat v1.12.0 R package^28^ and was processed with PICRUSt2^29^. EC number, KO metagenomes, as well as MetaCyc pathway abundances were predicted. Differences in MetaCyc pathway abundances between microbiotypes were further analyzed using the DESeq2 v1.24.0 R package^30^. A p-value of < 0.05 after Bonferroni’s multiple test correction was considered statistically significant.

## Results

### Subject characteristics

Seventy-three Argentinean subjects were enrolled in this study, who were classified as normal weight (n = 32), overweight (n = 12), or obese (n = 29). Group characteristics are shown in Table 1.

**Table 1:** Clinical characteristics of the Argentinean population.

N (f/m)	Lean			Overweight			Obese		
	N (f/m)	Age (year)	BMI (kg/m^2^)	N (f/m)	Age (year)	BMI (kg/m^2^)	N (f/m)	Age (year)	BMI (kg/m^2^)
73 (45/28)	32 (22/10)	40.2 [34.8–45.6]	22.5 [21.8–23.2]	12 (5/7)	41.5 [32.9–50.1]	27.4 [26.6–28.2]	29 (18/11)	49.1 [46.25–55.0]	33.2 [32.0–34.4]

*N* population size, *f* female, *m* male. Age and BMI expressed as mean and 95% CI.

### Alpha- and beta-diversity within and among Argentinean BMI groups

**Figure 1:**
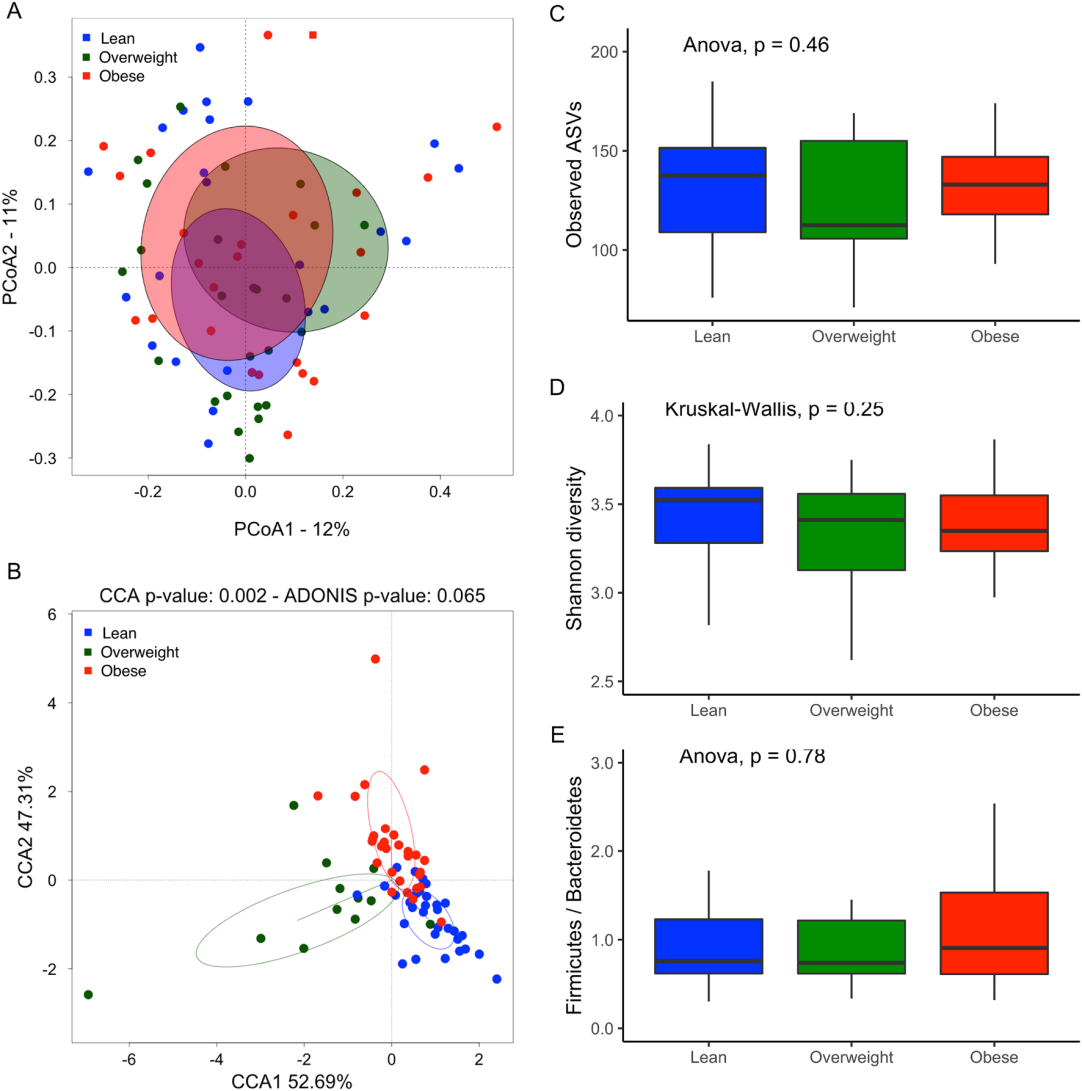
Analysis of the microbiota in the Argentinean population according to their BMI status. (**A**,**B**) Comparison of the microbiota profiles using the Principal Coordinates Analysis (PCoA) and the Canonical Correspondence Analysis method (CCA) based on the Bray–Curtis distance. The ellipses represent the standard deviation (**C**–**E**) Box-plots showing the Observed ASV diversity (**B**), the Shannon diversity (**C**), the Firmicutes/Bacteroidetes ratio (**D**). The solid black lines indicate medians, and the lower and upper bounds of the box represent the 25 and 75% quartiles. Outliers are indicated as black circles and represent samples falling outside the 10 and 90% quartiles. Statistical analysis was performed using the ANOVA test. Each color represents a specific BMI group population: blue box (Lean), green box (Overweight) and red box (Obese).

Comparative analysis revealed that beta-diversity significantly differs among the three BMI groups (Fig. 1A,B), indicating dissimilarities in the composition and taxa abundance of their microbiota. In contrast, alpha-diversity, which reflects the richness of gut bacterial communities and was measured as observed ASVs and Shannon index, did not differ among the three BMI groups (Fig. 1C,D).

### Taxa associated with obesity in Argentinean subjects

The relative abundance of bacterial taxa in the 3 BMI groups was compared by Kruskal–Wallis test (Supplementary data, Table S1). Though the abundance of the phyla Bacteroidetes and Firmicutes and the ratio Firmicutes/Bacteroidetes did not fluctuate (Fig. 1E), differences in some bacterial taxa were observed between BMI groups. Within the Bacteroidetes phylum, the abundances of Porphyromonadaceae and Rikenellaceae were significantly lower in the obese than in the normal weight subjects (p = 0.023 and p = 0.016, respectively; Supplementary data, Fig. S1), and correlated inversely with BMI (R = − 0.35, p = 0.0021 and R = − 0.37, p = 0.0015, respectively; Supplementary data, Fig. S2). When looking at lower taxa level, *Barnesiella* sp. from the Porphyromonadaceae family, *Alistipes shahii,* and *Alistipes* sp. from the Rikenellaceae family were significantly lower in obese subjects than in normal weight individuals (p < 0.040, p < 0.011, and p < 0.006, respectively). Two species of the Bacteroidaceae family, *B. caccae* and *B. dorei* were less abundant in the obese subjects (p = 0.011 and p = 0.026, respectively). Notably, overweight subjects also showed most of the differences observed in the obese group.

Various taxa of the Firmicutes phylum also differed in the obese individuals. Specifically, higher abundances of *Roseburia* (p = 0.006), *Ruminococcus* sp. (p = 0.042) and *Blautia producta* (p = 0.011) from the Lachnospiraceae family were detected. Furthermore, genera/species from Erysipelotrichaceae, Clostridiaceae, and Ruminococcaceae families such as *Eubacterium biforme* (p = 0.015), *Clostridium lactatifermentans* (p = 0.008) and *Ruminiclostridium* sp. (p = 0.007), were more abundant in the obese subjects. Interestingly, only the abundance of *Roseburia* was significantly higher in the overweight subjects compared to lean individuals. Remarkably, the abundances of other taxa such as *R. gnavus* (p = 0.040) and *Lactobacillus* (p = 0.004), mainly *L. rogosae* (p = 0.024) remained unaltered in obese subjects but were higher in the overweight group. Gracilibacteraceae (p = 0.002) and Peptococcaceae (p = 0.002) were significantly depleted in both overweight and obese subjects. Additionally, a lower abundance of *Oscillibacter* sp. (p = 0.048) from the family Oscillospiraceae, was observed in obese but not in overweight subjects.

Finally, lower proportions of the phyla Tenericutes (p = 0.007), represented by the Mollicutes class (p = 0.005), and Verrucomicrobia (p = 0.029) were observed in obese and overweight subjects. Noteworthy, the abundance of these two phyla inversely correlated with BMI (R = − 0.33, p = 0.0048 and R = − 0.37, p = 0.0011, respectively; Supplementary data, Fig. S2).

### Gut microbiota in obese subjects from different geographical populations

**Figure 2:**
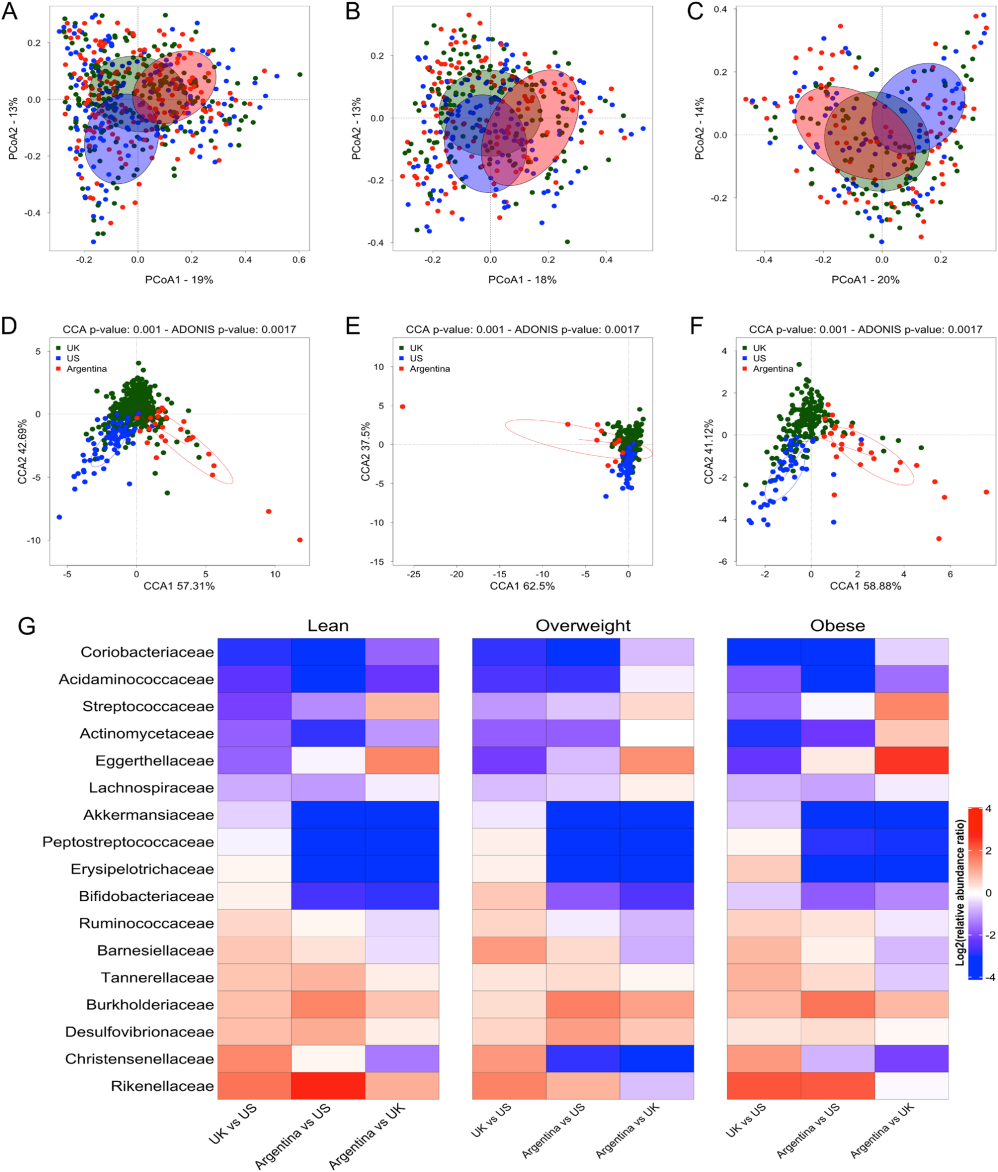
Differences in the microbiota composition among Argentinean, US and UK populations for each BMI group. (**A**–**F**) Comparison of microbiota profiles in Lean (**A**,**D**), Overweight (**B**,**E**) and Obese (**C**,**F**) using the Principal Coordinates Analysis (PCoA) (**A**–**C**) and the Canonical Correspondence Analysis method (CCA) (**D**–**F**) based on the Bray–Curtis distance. Each color represents a specific population (i.e. Argentine, UK and US). (**G**) Heat map representing the differential log2 (relative abundance ratio) changes of bacterial taxa common to the 3 groups, lean, overweight and obese, in populations of different geographic origins. Heatmap color (blue to dark red) displays the row scaled log2 (relative abundance ratio) of each taxon.

To detect specific features in the obesity associated gut microbiota structure, the bacterial community composition of obese Argentinean was compared to that of individuals from different geographic origins^18,19^. High-throughput sequences of the 16S rRNA gene corresponding to the V4 hypervariable region and clinical data from previous studies carried out in UK and US were collected (Table 2). Samples with less than 10,000 reads were removed by quality filtering, so that finally 63 Argentinean subjects were compared with 1,150 individuals from UK and US. These studies were selected based on the number of analyzed samples, free access to sequencing data and availability of individual clinical data (i.e. age, sex, BMI). To allow direct comparison among sequences from different studies, all the reads in the V4 hypervariable region were aligned and trimmed to the same length (100 bp), then analyzed using the DADA2 pipeline based in the identification of Exact Sequence Variants. Although it has been reported that a sequence length of 100 bp in the V4 region is adequate for the correct analysis of the microbial communities^31^, we limited the identification of reads to the level of family/genus, the rate of identification of lower level taxa being not appropriate. For each BMI group the similarity in overall bacterial composition among individuals from the three countries was determined using Principal Coordinates Analysis (PCoA) (Fig. 2A–C) and Canonical Correspondence Analysis (CCA) (Fig. 2D–F). As shown in these graphics, the gut microbiota of the lean, overweight and obese subjects clustered separately according to the geographic origin of the subjects (Fig. 2D–F), indicating significant differences in their microbiota composition. The analysis revealed that 25 bacterial families exhibited different abundances between BMI groups, (Supplementary data Fig. S3), 17 of them differed significantly when comparing between countries within each BMI Group. To investigate whether the changes in these bacterial taxa were dependent or independent of the BMI status, we compared the countries two by two in each BMI group and calculated the log_2_ (relative abundance ratio) for these Families (Fig. 2G). Bacterial differences were considered as independent of the BMI status whether similar changes were observed for the same countries in all BMI groups. Results showed that the increase/decrease log_2_(foldchange) were similar for 8/17 families: Actinomycetaceae, Streptococcaceae, Barnesiellaceae, Erysipelotrichaceae, Acidaminococcaceae, Akkermansiaceae, Coriobacteriaceae and Burkholderiaceae. For example, in the lean group, the microbiota of the English subjects was impoverished in Coriobacteriaceae compared with that of the Americans, and these differences persisted in the overweight and obese subjects from both countries. In contrast, some discrepancies in the overweight or obese groups were noted for the others 9/17 families, although the global profiles of increased/decreased log_2_(relative abundance ratio) among BMI groups were close. Together, these data indicate that the geographical origin of the subjects affects in a comparable way the composition of their gut microbiome, independently of the BMI status.

**Table 2:** Description of studies considered to compare geographical population.

Country	Study	Accession number	N (f/m)	Lean			Overweight			Obese		
				N (f/m)	Age (year)	BMI (kg/m^2^)	N (f/m)	Age (year)	BMI (kg/m^2^)	N (f/m)	Age (years)	BMI (kg/m^2^)
US	^18^	PRJNA290926	172 (111/61)	68 (49/19)	53.1 [50.5–55.7]	22.0 [21.5–22.5]	57 (31/26)	55.1 [53.2–57.0]	27.1 [26.7–27.4]	47 (31/16)	55.0 [51.6–58.4]	35.0 [33.1–34.0]
UK	^19^	PRJEB6702	507 (NI)	230 (NI)	61.2 [59.9–62.5]	22.3 [22.1–22.6]	174 (NI)	62.9 [61.7–64.1]	27.3 [26.9–27.1]	103 (NI)	59.7 [58.2–61.2]	34.0 [33.3–34.7]
UK	^19^	PRJEB6705	471 (NI)	220 (NI)	60.0 [58.8–61.3]	22.3 [22.0–22.5]	162 (NI)	62.7 [59.5–61.1]	27.2 [27.0–27.5]	89 (NI)	60.2 [58.2–62.2]	34.6 [33,0–33.8]
Argentine	This study	This study	63 (41/22)	28 (20/8)	39.0 [33.3–44.73]	22.3 [21.5–23.1]	11 (4/7)	41.0 [31.6–50.5]	27.4 [26.5–28.3]	24 (17/7)	49.1 [44.3–54.0]	33.2 [31.7–34.6]

*N* population size, *f* female, *m* male. Age and mean expressed as mean and 95% CI. *NI* not informed.

### Obese from distinct geographic population present different taxonomic signature

**Figure 3:**
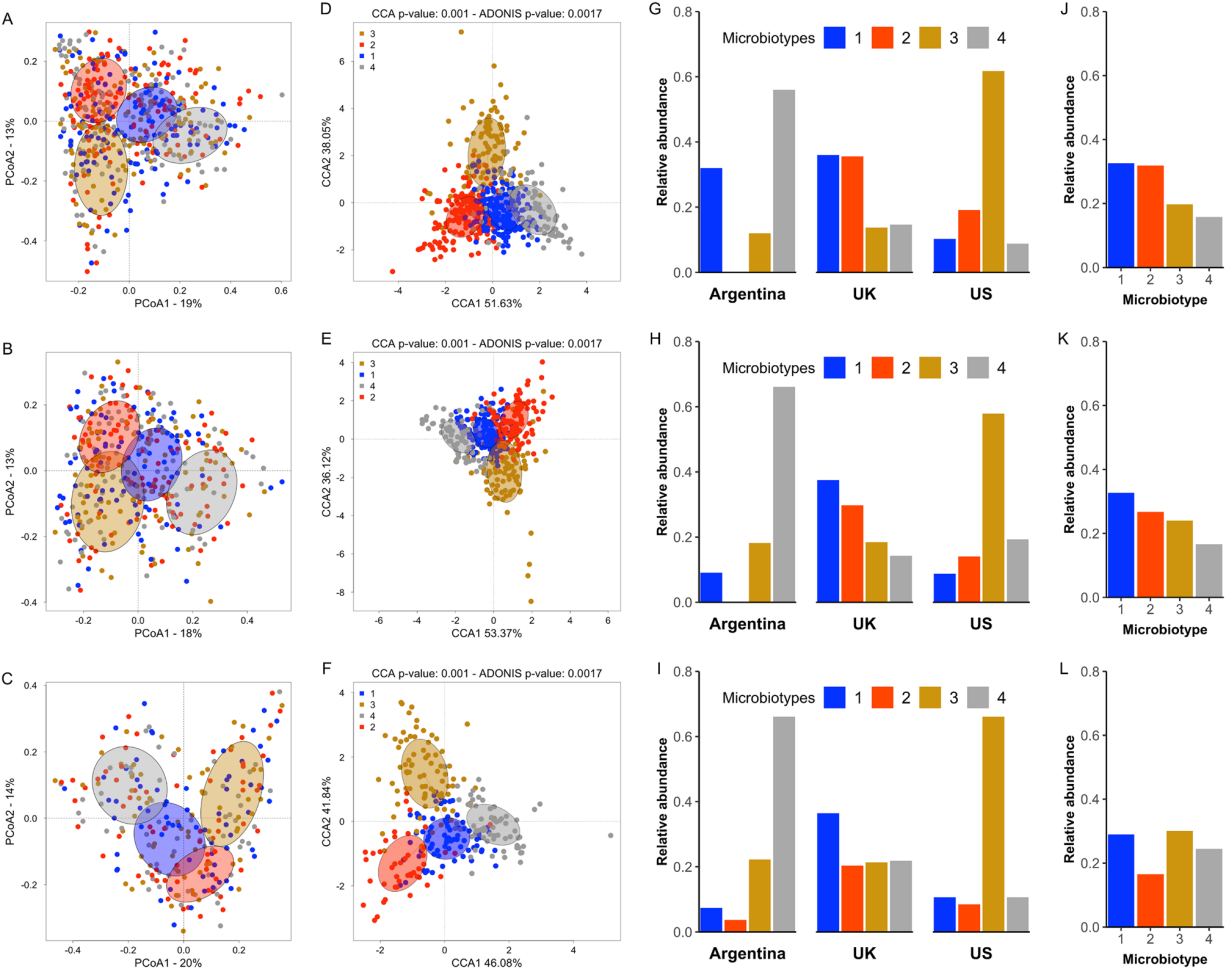
Analysis of microbiotypes distribution in the different subject population according their BMI status. (**A**–**F**) Clustering of the microbiotypes according to the distinct population in Lean (**A**,**D**), Overweight (**B**,**E**) and Obese (**C**,**F**) groups using the Principal Coordinates Analysis (PCoA) (**A**–**C**) and the Canonical Correspondence Analysis method (CCA) (**D**–**F**) based on the Bray–Curtis distance. (**G**–**I**) Distribution of Microbiotypes inside the distinct populations in Lean (**G**), Overweight (**H**) and Obese (**I**) groups. (**J**–**L**) Total distribution of Microbiotypes in Lean (**J**), Overweight (**K**) and Obese (**L**) groups. Each color represents a Microbiotype (i.e. Microbiotype-1, -2, -3 and -4).

**Figure 4:**
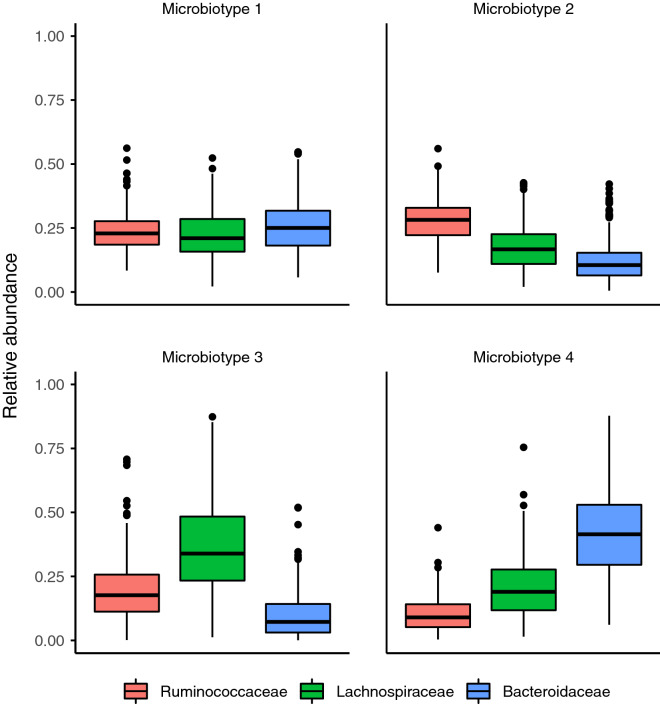
Abundance of the main families involved in the building of Microbiotypes. Boxplot representing the abundances of the Ruminococcaceae (red box), Lachnospiraceae (green box) and Bacteroidaceae (blue box) in the Microbiotype-1 (**A**), -2 (**B**), -3 (**C**) and -4 (**D**). The solid black lines indicate medians, and the lower and upper bounds of the box represent the 25 and 75% quartiles. Outliers are indicated as black circles and represent samples falling outside the 10 and 90% quartiles.

**Figure 5:**
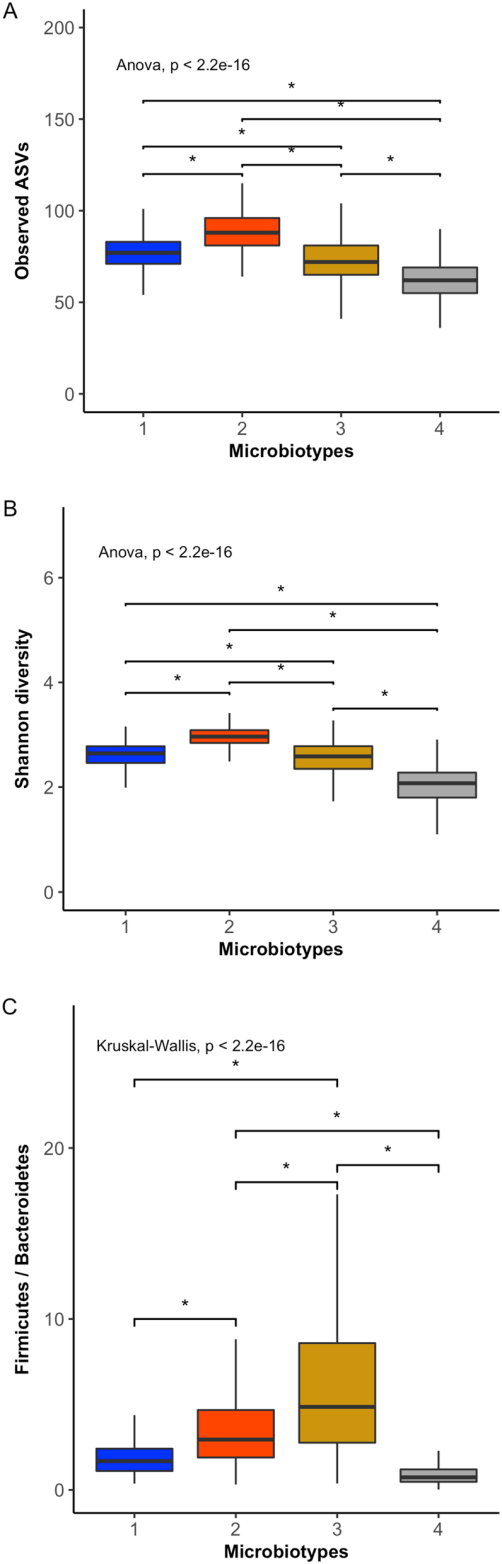
Ecological characteristic of the distinct microbiotypes. Box-plots show the Observed ASV diversity (**A**), Shannon diversity (**B**), Firmicutes/Bacteroidetes ratio (**C**) for each microbiotypes. The solid black lines indicate medians, and the lower and upper bounds of the box represent the 25 and 75% quartiles. Statistical differences (*) between microbiotypes were determined using ANOVA or Kruskal Wallis test supported by Tukey or Dunn test respectively (p < 0.05).

A probabilistic method, the Dirichlet Multinomial Mixtures (DMM), was applied to determine whether the microbiota of the obese subjects from the three countries might be stratified in specific microbial communities. DMM is generally used for clustering microbial community data by examining the frequency of bacterial taxa and determining the number of “metacommunities” or microbial community states (MCS) present in the dataset. Based on BIC approximation, which specifies that a lower value indicates a better fitting model, four distinct microbiotypes were identified in the 3 BMI groups (Fig. 3A–F). Taxa contributing to the microbiotypes are presented in Supplementary data, Table S2. Three bacterial Families (Ruminococcaceae, Lachnospiraceae and Bacteroidaceae) determined the difference between the four microbiotypes (Fig. 4). While none of these families was dominant in Microbiotype-1, Microbiotype-2 was dominated by Ruminococcaceae, Microbiotype-3 by Lachnospiraceae and Microbiotype-4 by Bacteroidaceae. Interestingly, the alpha-diversity and Firmicutes/Bacteroidetes ratio significantly differed among the four microbiotypes (Fig. 5A–C). Alpha-diversity (Observed ASVs and Shannon Index) was higher in Microbiotype-1 and -2 compared to Microbiotype-3 and -4 (Fig. 5A,B). The Firmicutes/Bacteroidetes ratio was < 1 for Microbiotype-4 and > 1 for Microbiotype-1, -2 and -3. Moreover, it was higher in Microbiotype-3 compared to the others (Fig. 5C).

On the other hand, the prevalence of the microbiotypes differs according to the country (Fig. 3G–I). When considering healthy subjects, Microbiotype-4 was more frequent in the Argentineans, Microbiotype-3 in the Americans and Microbiotype-1 and -2 among the English (Fig. 5A). Nevertheless, when all individuals from these countries were taken together and stratified according their BMI, the prevalence of Microbiotype-3 and -4 was higher in the obese groups compared to lean groups (19.7% vs*.* 30.1%; 15.8% vs*.* 24.4% respectively) while the prevalence of Microbiotype-2 was lower (31.9% vs*.* 16.5%) (χ^2^ = 32.8, p = 0.000011) (Fig. 3J–L).

### Microbiota functionality differs between microbiotypes

**Figure 6:**
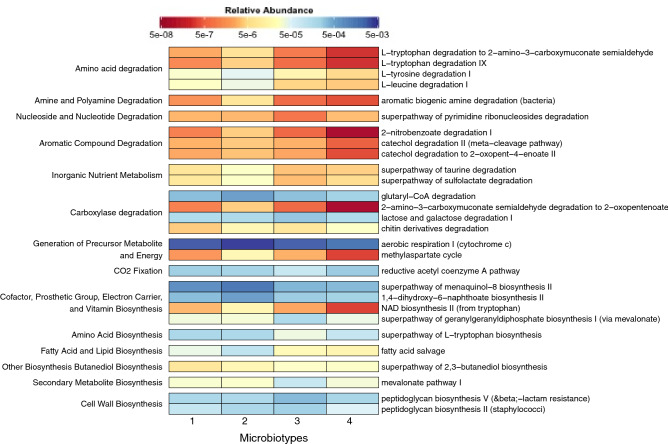
Prediction of the metagenomic functions in the different microbiotypes. Heatmap representing the metabolic pathways, predicted using PICRUSt2, in the four microbiotypes, higher and lower than 1.5 fold changes statistically different [DESeq2 (FDR < 0.05)].

To explore the metabolic pathways associated to the different microbiotypes, we carried out a metagenome functional prediction using PICRUSt2^27^. For this analysis, we considered the 20 bacterial families previously identified as the main contributors to the different microbiotypes. This approach allowed the identification of 362 metabolic pathways whose abundances among the four microbiotypes were compared using the R DESeq2 package. Multiple separate tests were performed on the metabolic pathways showing log_2_(Fold Change) lower or upper than 1.5. As depicted in Fig. 6, twenty-eight metabolic pathways significantly altered in at least one of the microbiotypes were identified.

Microbiotype-1 exhibited similar abilities than Microbiotype-2; although in lesser extent. In general, they were characterized by an improved capacity of degradation and biosynthesis compared to the Microbiotype-3 and 4, with higher capacity of amino acids degradation (Tryptophan, Tyrosine and Leucine) as well as amine compounds, nucleosides and nucleotides, aromatic and organic compounds (taurine and sulfolactate). Additionally, carboxylase degradation pathway were also increased (i.e. glutaryl-CoA and 2-amino-3-carboxymuconate semialdehyde). Consistently, these degradation pathways were associated with a better capacity of energy production (i.e. aerobic respiration and methylaspartate cycle).

On the other hand, Microbiotype-3 and -4 exhibited lower capacity of protein degradation (i.e. amino acids, aromatic compounds, taurine and sulfolactate), increased abundance of pathways involved in the metabolism of lactose/galactose and chitin derivatives, and decreased ability of quinol, quinone, fatty acids and lipids synthesis. Compared to the others, Microbiotypes 3 and 4 seem to have particularly increased biosynthetic pathways, including those implicated in the synthesis of a variety of terpenes, terpenoids, and 2,3-butanediol through the super-pathways of geranylgeranyl diphosphate and 2,3-butanediol, respectively. Finally, is important to remark that Microbiotype-3 showed increased abundance of pathways involved in the biosynthesis of peptidoglycan and mevalonate, but was deficient in those linked to l-tryptophan biosynthesis.

## Discussion

The aim of this study was to characterize the gut microbiota of Argentinean individuals with different nutritional status using 16SrRNA gene sequencing. Our results confirm that increased BMI is associated with alterations in gut microbiota composition. The obese and overweight Argentinean subjects exhibited significant differences in the abundance of various bacterial taxa, compared with the lean individuals. Thus, the families Rikenellaceae, Bacteroidaceae, Porphyromonadaceae (Bacteroidetes phylum), Gracilibacteraceae and Peptococcaceae (Firmicutes phylum) were decreased, while the Lachnospiraceae, Erysipelotrichaceae, Clostridiaceae and Ruminococcaceae families (Firmicutes Phylum) increased. Interestingly, several populations of butyrate-producing bacteria including *Alistipes*, *Oscillibacter* and *Faecalibacterium*, and members of the phylum Verrucomicrobia, whose main representant in the human colon is *Akkermansia muciniphila,* were less abundant in the obese group, confirming previous studies in other populations. Some authors have described an association among obesity, lower bacterial diversity and higher Firmicutes/Bacteroidetes ratio^6,32,33^. In contrast, negative correlations among microbiota diversity and BMI, body fat content, dyslipidemia, impaired glucose homeostasis and low-grade inflammation have also been described^7,8,34^. Nevertheless, several studies did not confirm these results or reported contrary findings, so the use of bacterial richness and Firmicutes/Bacteroidetes ratio as marker of obesity is currently excluded^6,35–41^. Consistent with these results, our study did not observe any differences in alpha diversity and Firmicutes/Bacteroidetes ratio in the gut microbiota of the overweight and obese groups, compared with the lean. As described elsewhere, it is therefore probable that other factors including geographical location, physical activity, lifestyle, cultural and dietary habits, increase the variability of the Firmicutes/Bacteroidetes ratio in healthy subjects^17,42–45^.

Our second aim was to compare the gut microbiota composition of obese and overweight Argentinean subjects with the microbiota structure from UK and US individuals. A limitation in the comparison of sequencing data from different studies is that it is influenced by several factors including sample processing, DNA extraction method, primer selection, sequencing method, and bioinformatic analysis^45^. It is therefore challenging to consider all these sources of bias that can result in the over- or under-representation of individual taxa within the bacterial community. To overcome this limitation, all reads were re-analyzed using a unique pipeline (DADA2), then aligned and trimmed to the same length (100pb) for limiting bias in the assignment of the taxonomy and allow direct comparisons among sequences from different studies. Our analysis revealed that gut microbiota composition differed according the country, not only for healthy individuals but also for overweight and obese subjects. The taxa involved in these differences were similar when results were compared by BMI group. Among the 17 bacterial families whose abundance significantly differed between countries, eight increased or decreased in a similar way in the three BMI groups. These data indicate that the microbiota composition of lean, overweight and obese people changes in the same way depending on their geographical location. Then the geographical location could act as an environmental factor driving the composition of the obese gut microbiota. However, the fact that some changes in bacterial family abundances differ in the BMI groups suggests that other factors probably interfere, that have not been considered or identified in the three studies currently analyzed.

Although numerous factors have been shown to affect the mammalian gut microbiota composition, dietary habits have been reported as the most important in shaping the diversity of the community^46–48^. Even if dietary intake was not determined in the obese individuals participating in these 3 studies, it probably explains part of the differences observed among the obese groups of the different geographical populations^49,50^. Additionally, metabolic complications such as insulin resistance, fatty liver disease and low-grade inflammation might also influence the differences in the gut microbiome of obese among the different populations. However, our study cannot confirm this hypothesis, as clinical and nutritional data were not registered.

Subsequently, we determined that BMI status was associated with specific microorganism consortia (defined as microbiotype in our study) rather than individual bacterial taxa. According to the geographic origin of the subjects, gut microbiota clustered in four microbiotypes, harboring mainly three different bacterial families: Ruminococcaceae, Lachnospiraceae and Bacteroidaceae representing the most dominant taxa, whose abundance is probably related to dietary intake. Ruminococcaceae and Lachnospiraceae enrichment has been previously associated with high fat diets while high intakes of carbohydrates and fibers were also associated with increases of these same families accompanied by decreased Bacteroidaceae^51^. Although Bacteroidaceae are capable of degrading various plant polysaccharides, human studies showed they do not respond effectively to fiber supplementation^52^. Indeed, the fermentative activity of SCFA-producing bacteria, mostly represented by Ruminococcaceae and Lachnospiraceae (from the Firmicutes phylum), reduces the colonic pH, inhibiting the growth of acid-sensible bacteria such as the Bacteroidaceae, and increasing acid-tolerant bacteria^51^. On the other hand, the growth of bacteria from the Bacteroidaceae family is favored by high-protein, low fermentable fiber diets^51^. Ruminococcaceae, Lachnospiraceae and Bacteroidaceae were previously shown to discriminate populations according to their diet^49,53–55^. In contrast to other studies, we did not identify the genus Prevotella as a key determinant of microbiota structure^49,53–56^. Although *Prevotella* is an important fiber degrader, it has been mainly identified as a member of the microbiota of non-Westernized populations^49,53–55^, not included in our study. Interestingly, in Western populations, the abundance of Prevotella correlates negatively with fiber intake^56^. Therefore, it appears that diet could contribute in the establishment of the microbiotypes identified in our study, acting as a driver of inter-individual microbiota differences.

A microbiotype can be considered as a functional, harmonious, association of several bacterial species driven by host dietary habits and nutritional status. Indeed, a positive relation was observed between microbiotypes and BMI groups. Likewise, Microbiotype-1 which exhibits similar abundance of the three bacterial families, and Microbiotype-2 with higher abundance of Ruminococcaceae, harbored higher bacterial diversity and were more abundant in the UK population, compared to other microbiotypes. The frequency of both Microbiotypes -1 and -2 was lower in overweight and obese subjects. This finding confirm a previous study reporting that gut microbiota with high diversity and enriched in Ruminococcaceae and Lachnospiraceae were associated with lower weight gain at long-term^57^. Microbiotype-3 displayed higher abundance of Lachnospiraceae and was more represented in the US subjects while Microbiotype-4, enriched in Bacteroidaceae, was more representative of the Argentinean subjects, what could be explained by their habitual meat-oriented diet. Additionally, Microbiotype-3 and -4, which had low bacterial diversity and high Firmicutes/Bacteroidetes ratio, were more abundant in overweight and obese individuals. An increased risk of weight gain has been associated with low diversity (as in Microbiotype-3 and -4), and enrichment in Bacteroides species (as in Microbiotype-4)^57^. Together these data support that the composition of the microbiota results from a close interaction between diet and BMI status, making difficult to define a specific obesity-associated microbiota signature.

Our study also explored the metabolic activities of the different microbiotypes through PICRUST2 which predicts metabolic pathways based on 16S rRNA data. PICRUST only predicts the portion of the metagenome corresponding to the set of microorganisms identified in our study. Whether a bacterial taxon is not identified, due to misamplification for example, its contribution will not be predicted. Another limitation is that this strategy only predicts gene families already known and included in the reference database. Therefore, bacterial genes not included in the database will not be predicted, despite their potential importance in the ecosystem^27^. Considering these limitations, PICRUST2 analysis attributed distinct functional profiles to the different microbiotypes. Compared to other microbiotypes, microbiotypes 3 and 4 seem to have a lower capacity of protein degradation, which might be a contradictory result since the microbiotype 4 was predominant in Argentinean population known by the intake of a high-meat diet. Even though individual food consumption surveys were not available, it is known that dietary polyunsaturated fatty acid intake could reduce the protein fermentation. In fact, it has been shown that dietary polyunsaturated fatty favor the growth of Lachnospiraceae, as they can degrade these compounds^58,59^. Accordingly, some specific dietary components, such as fatty acids, can influence the microbial metabolism of other components. These results might explain the lower capacity of protein degradation observed in the microbiotype 3.

Taken together, these observations suggest that diet shapes the composition and functionality of the gut microbiota not only in healthy subjects but also in obese. There is not a unique microbiota structure associated with obesity but a set of microbiota differing in their composition. The contribution of the microbiome to obesity is determined through interactions among different factors including geographical, dietary, genetic and environmental. Indeed, the type of diet that shapes microbiota composition might additionally contribute to the pathological functioning of the obesogenic microbiome. Although these results need to be confirmed, they may explain the discrepancies in the microbiome composition of obese reported in a number of studies^48,49,57^.

## Conclusion

As shown in the Argentinean population, it is undeniable that gut dysbiosis is associated with obesity; our study also revealed that geographical factors affect the composition of obese microbiota, preventing the identification of a global taxonomic signature. Indeed, the analysis of different geographical populations suggests that obesity associated dysbiosis would comprise several gut microbiota structures. In addition, we showed that the microbiota structure is determined in part by the abundance levels of Ruminococcaceae, Lachnospiraceae and Bacteroidaceae, which might be an important factor of human health. Identification of distinct taxonomic signatures and the metabolic pathways associated with obesity may be a novel promising tool to stratify patients based on their microbiome configuration to design treatment or prevention strategies focusing gut microbiota manipulation for obesity management.

## Supplementary Information

Supplementary Information.
