# Supplementary material for: Comparison of Argentinean microbiota with other geographical populations reveals different taxonomic and functional signatures associated with obesity
Source: Sci Rep. 2021 Apr 8;11:7762. doi: 10.1038/s41598-021-87365-x (PMC8032766; doi:10.1038/s41598-021-87365-x)
Supplement: Supplementary file 1 — Supplementary Information. [file 41598_2021_87365_MOESM1_ESM.pdf]

Comparison of Argentinean microbiota with other geographical populations reveals different taxonomic and functional signature associated with obesity

Susana Pesoa[1*], Nestor Portela[1], Eduardo Fernández[1], Osvaldo Elbarcha[1], Martin Gotteland[2,3], Fabien Magne[4*]

[1] Department of Molecular Diagnosis, LACE Laboratories, Córdoba, Argentina.

[2]Department of Nutrition, Faculty of Medicine, University of Chile.

[3]Institute of Nutrition and Food Technology (INTA), University of Chile.

[4]Microbiology and Mycology Program, Biomedical Sciences Institute (ICBM), School of Medicine, University of Chile.

Keywords: Microbiota; Gut; Obesity; Dysbiosis; Argentina

*Corresponding author: Fabien Magne, PhD.

Microbiology and Mycology Program,

Biomedical Sciences Institute (ICBM),

School of Medicine, University of Chile,

Av. Independencia 1027, Independencia, Santiago, Chile.

fabienmagne@med.uchile.cl

Tel.: +56 2 2978 9627

*Corresponding author: Susana A Pesoa, PhD

Department of Molecular Diagnosis

LACE Laboratories

Avda Velez Sarsfield 528, Córdoba, Argentina

susana.pesoa@laboratoriolace.com.ar

Tel.: +54 351 4246666. Int. 143

<u>Supplementary data Figure S1:</u> Heatmap of the relative abundance of bacterial taxa in the Overweight and Obese groups, compared to the Lean group in the microbiota of the Argentinean subjects. Heatmap color (blue to dark red) displays the row scaled log2(fold change) of each taxon. * significant differences established using the Kruskal Wallis test and Dunn post-hoc test to evaluate statistical differences between groups ($p<0.05$).

<u>Supplementary data Figure S2:</u> Significant correlations between body mass index (BMI) and microbial taxa.

<u>Supplementary data Figure S3:</u> Heatmap representing all differentially log2(relative abundance ratio) of bacterial taxa between the distinct population, identified in Lean, Overweight and Obese groups. Heatmap color (blue to dark red) displays the row scaled log2(relative abundance ratio) of each taxon. * shows relative abundance of taxa significantly different between the countries (Kruskal–Wallis test) in the BMI group corresponding.

<u>Supplementary data Table S1:</u> Relative abundances of taxa observed in the fecal microbiota of Argentinian subjects according to their BMI status. Only taxa present in at least 16 subjects of Lean group or 6 of overweight group and 14 of obese group were presented. p values in bold style were statistically significant.

<u>Supplementary data Table S2:</u> Relative abundance of the bacterial families determining the four microbiotypes identified in the study. Their relative abundance was calculated by dividing the abundance of the taxa by the abundance of the total number of other taxa identified in this study. NC: Taxa not contributing in the microbiotype.

Figure S1

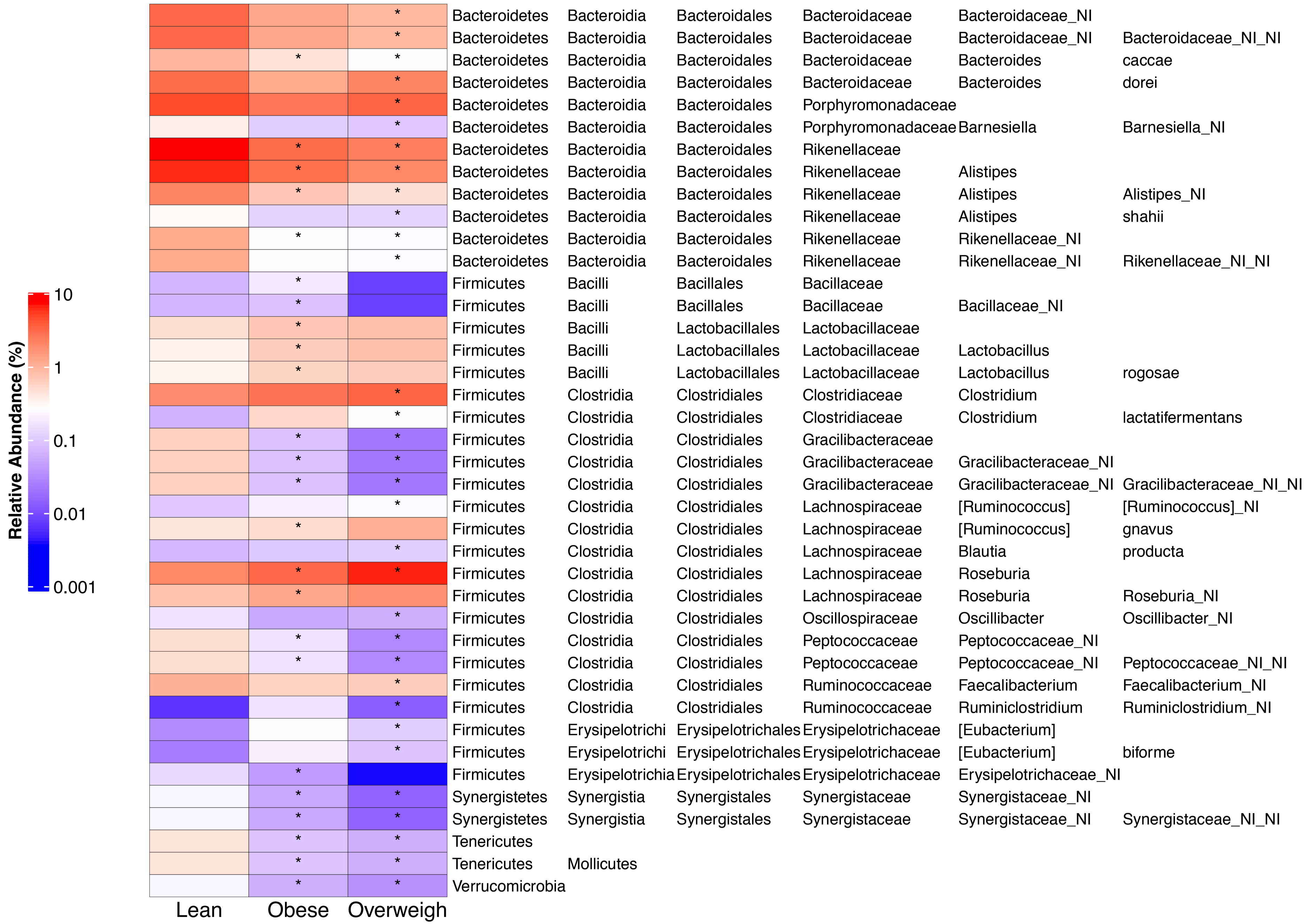
Relative Abundance (%)
10
1
0.1
0.01
0.001
Lean
Obese
Overweigh
Bacteroidetes Bacteroidia Bacteroidales Bacteroidaceae Bacteroidaceae_NI
Bacteroidetes Bacteroidia Bacteroidales Bacteroidaceae Bacteroidaceae_NI Bacteroidaceae_NI_NI
Bacteroidetes Bacteroidia Bacteroidales Bacteroidaceae Bacteroides caccae
Bacteroidetes Bacteroidia Bacteroidales Bacteroidaceae Bacteroides dorei
Bacteroidetes Bacteroidia Bacteroidales Porphyromonadaceae
Bacteroidetes Bacteroidia Bacteroidales Porphyromonadaceae Barnesiella Barnesiella_NI
Bacteroidetes Bacteroidia Bacteroidales Rikenellaceae
Bacteroidetes Bacteroidia Bacteroidales Rikenellaceae Alistipes
Bacteroidetes Bacteroidia Bacteroidales Rikenellaceae Alistipes Alistipes_NI
Bacteroidetes Bacteroidia Bacteroidales Rikenellaceae Alistipes shahii
Bacteroidetes Bacteroidia Bacteroidales Rikenellaceae Rikenellaceae_NI
Bacteroidetes Bacteroidia Bacteroidales Rikenellaceae Rikenellaceae_NI Rikenellaceae_NI_NI
Firmicutes Bacilli Bacillales Bacillaceae
Firmicutes Bacilli Bacillales Bacillaceae Bacillaceae_NI
Firmicutes Bacilli Lactobacillales Lactobacillaceae
Firmicutes Bacilli Lactobacillales Lactobacillaceae Lactobacillus
Firmicutes Bacilli Lactobacillales Lactobacillaceae Lactobacillus rogosae
Firmicutes Clostridia Clostridiales Clostridiaceae Clostridium
Firmicutes Clostridia Clostridiales Clostridiaceae Clostridium lactatifermentans
Firmicutes Clostridia Clostridiales Gracilibacteraceae
Firmicutes Clostridia Clostridiales Gracilibacteraceae Gracilibacteraceae_NI
Firmicutes Clostridia Clostridiales Gracilibacteraceae Gracilibacteraceae_NI Gracilibacteraceae_NI_NI
Firmicutes Clostridia Clostridiales Lachnospiraceae [Ruminococcus] [Ruminococcus]_NI
Firmicutes Clostridia Clostridiales Lachnospiraceae [Ruminococcus] gnavus
Firmicutes Clostridia Clostridiales Lachnospiraceae Blautia producta
Firmicutes Clostridia Clostridiales Lachnospiraceae Roseburia
Firmicutes Clostridia Clostridiales Lachnospiraceae Roseburia Roseburia_NI
Firmicutes Clostridia Clostridiales Oscillospiraceae Oscillibacter Oscillibacter_NI
Firmicutes Clostridia Clostridiales Peptococcaceae Peptococcaceae_NI
Firmicutes Clostridia Clostridiales Peptococcaceae Peptococcaceae_NI Peptococcaceae_NI_NI
Firmicutes Clostridia Clostridiales Ruminococcaceae Faecalibacterium Faecalibacterium_NI
Firmicutes Clostridia Clostridiales Ruminococcaceae Ruminiclostridium Ruminiclostridium_NI
Firmicutes Erysipelotrichi Erysipelotrichales Erysipelotrichaceae [Eubacterium]
Firmicutes Erysipelotrichi Erysipelotrichales Erysipelotrichaceae [Eubacterium] biforme
Firmicutes Erysipelotrichia Erysipelotrichales Erysipelotrichaceae Erysipelotrichaceae_NI
Synergistetes Synergistia Synergistales Synergistaceae Synergistaceae_NI
Synergistetes Synergistia Synergistales Synergistaceae Synergistaceae_NI Synergistaceae_NI_NI
Tenericutes
Tenericutes Mollicutes
Verrucomicrobia

Figure S2

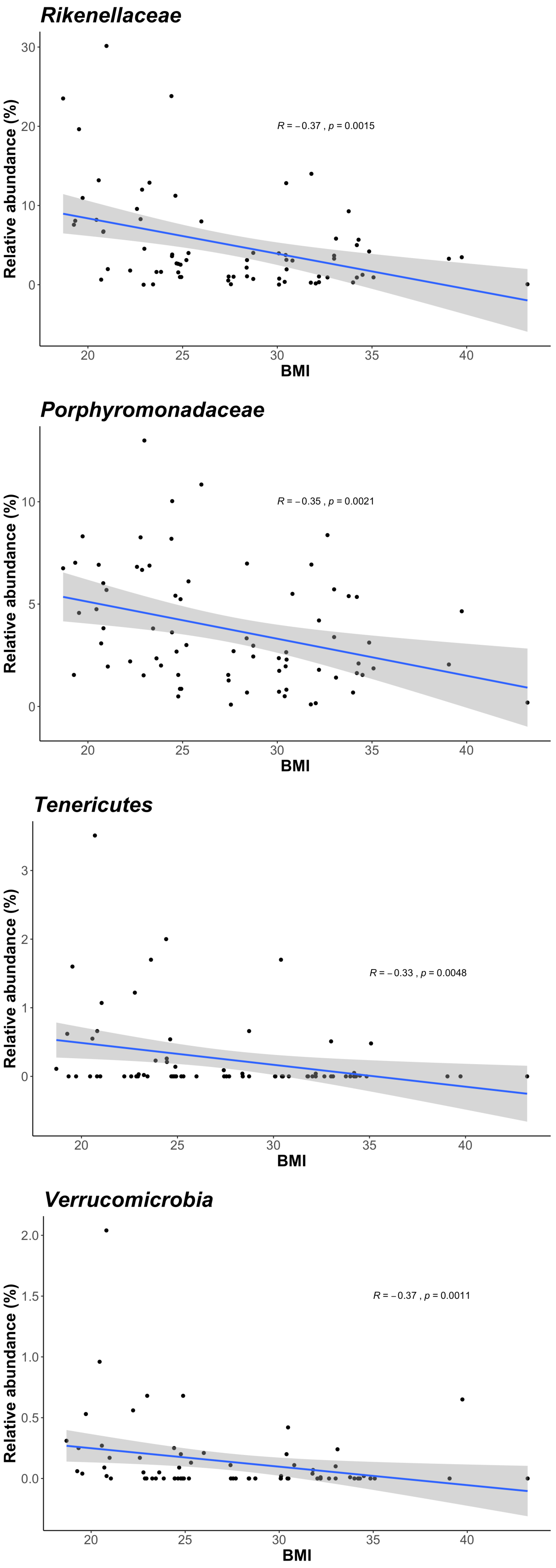
Rikenellaceae
R = −0.37 , p = 0.0015
Relative abundance (%)
BMI
Porphyromonadaceae
R = −0.35 , p = 0.0021
Relative abundance (%)
BMI
Tenericutes
R = −0.33 , p = 0.0048
Relative abundance (%)
BMI
Verrucomicrobia
R = −0.37 , p = 0.0011
Relative abundance (%)
BMI

Figure S3

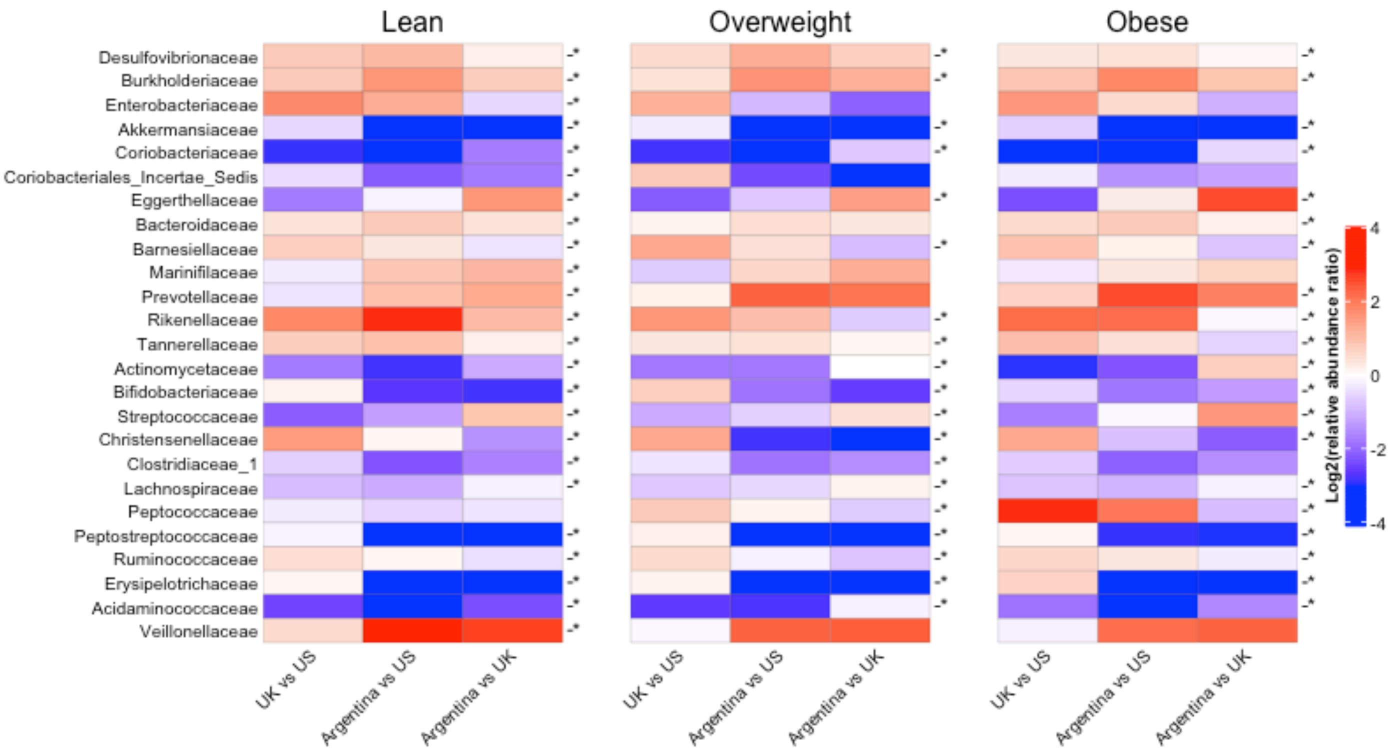

Lean
Overweight
Obese
Desulfovibrionaceae
Burkholderiaceae
Enterobacteriaceae
Akkermansiaceae
Coriobacteriaceae
Coriobacteriales_Incertae_Sedis
Eggerthellaceae
Bacteroidaceae
Barnesiellaceae
Marinifilaceae
Prevotellaceae
Rikenellaceae
Tannerellaceae
Actinomycetaceae
Bifidobacteriaceae
Streptococcaceae
Christensenellaceae
Clostridiaceae_1
Lachnospiraceae
Peptococcaceae
Peptostreptococcaceae
Ruminococcaceae
Erysipelotrichaceae
Acidaminococcaceae
Veillonellaceae
UK vs US
Argentina vs US
Argentina vs UK
Log2(relative abundance ratio)
4
2
0
-2
-4

Supplementary data Table S1:

Phylum	Class	Order	Family	Genus	Specie	Prevalence Leans	Mean Leans	SD Leans	Prevalence Overweights	Mean Overweights	SD Overweights	Prevalence Obeses	Mean Obeses	SD Obeses	p.values
Actinobacteria						32	1e-02	2e-02	11	7e-03	8e-03	28	1e-02	2e-02	0.674
Actinobacteria	Actinobacteria					32	9e-03	2e-02	11	6e-03	7e-03	28	1e-02	2e-02	0.568
Actinobacteria	Actinobacteria	Actinomycetales				9	4e-04	2e-03	5	1e-04	3e-04	8	9e-05	2e-04	0.479
Actinobacteria	Actinobacteria	Bifidobacteriales				24	5e-03	1e-02	7	2e-03	4e-03	16	5e-03	2e-02	0.225
Actinobacteria	Actinobacteria	Bifidobacteriales	Bifidobacteriaceae			24	5e-03	1e-02	7	2e-03	4e-03	16	5e-03	2e-02	0.225
Actinobacteria	Actinobacteria	Bifidobacteriales	Bifidobacteriaceae	Bifidobacterium		24	5e-03	1e-02	7	2e-03	4e-03	15	5e-03	2e-02	0.182
Actinobacteria	Actinobacteria	Bifidobacteriales	Bifidobacteriaceae	Bifidobacterium	adolescentis	13	4e-04	7e-04	5	4e-04	1e-03	10	2e-03	8e-03	0.988
Actinobacteria	Actinobacteria	Bifidobacteriales	Bifidobacteriaceae	Bifidobacterium	Bifidobacterium_NI	24	2e-03	8e-03	6	1e-03	2e-03	15	2e-03	7e-03	0.154
Actinobacteria	Actinobacteria	Coriobacteriales				27	4e-03	6e-03	10	4e-03	5e-03	27	8e-03	9e-03	0.248
Actinobacteria	Actinobacteria	Coriobacteriales	Coriobacteriaceae			27	4e-03	6e-03	10	4e-03	5e-03	27	8e-03	9e-03	0.248
Actinobacteria	Actinobacteria	Coriobacteriales	Coriobacteriaceae	Collinsella		24	1e-03	1e-03	7	2e-03	4e-03	22	3e-03	6e-03	0.270
Actinobacteria	Actinobacteria	Coriobacteriales	Coriobacteriaceae	Collinsella	aerofaciens	24	1e-03	1e-03	7	2e-03	4e-03	21	3e-03	6e-03	0.348
Actinobacteria	Actinobacteria	Coriobacteriales	Coriobacteriaceae	Coriobacteriaceae_NI		22	2e-03	3e-03	5	5e-04	9e-04	23	2e-03	3e-03	0.109
Actinobacteria	Actinobacteria	Coriobacteriales	Coriobacteriaceae	Coriobacteriaceae_NI	Coriobacteriaceae_NI_NI	22	2e-03	3e-03	5	5e-04	9e-04	23	2e-03	3e-03	0.066
Actinobacteria	Actinobacteria	Coriobacteriales	Coriobacteriaceae	Slackia		12	1e-03	3e-03	7	9e-04	1e-03	15	2e-03	3e-03	0.218
Actinobacteria	Actinobacteria	Coriobacteriales	Coriobacteriaceae	Slackia	isoflavoniconvertens	12	1e-03	2e-03	7	9e-04	1e-03	14	2e-03	3e-03	0.337
Actinobacteria	Coriobacteriia					21	6e-04	8e-04	8	1e-03	2e-03	20	1e-03	2e-03	0.517
Actinobacteria	Coriobacteriia	Coriobacteriales				21	6e-04	8e-04	8	1e-03	2e-03	20	1e-03	2e-03	0.417
Actinobacteria	Coriobacteriia	Coriobacteriales	Coriobacteriaceae			21	6e-04	8e-04	8	1e-03	2e-03	20	1e-03	2e-03	0.417
Actinobacteria	Coriobacteriia	Coriobacteriales	Coriobacteriaceae	Coriobacteriaceae_NI		21	5e-04	8e-04	8	1e-03	2e-03	19	1e-03	2e-03	0.542
Actinobacteria	Coriobacteriia	Coriobacteriales	Coriobacteriaceae	Coriobacteriaceae_NI	Coriobacteriaceae_NI_NI	21	5e-04	8e-04	8	1e-03	2e-03	19	1e-03	2e-03	0.552
Bacteroidetes						32	4e-01	2e-01	12	4e-01	2e-01	29	4e-01	2e-01	0.517
Bacteroidetes	Bacteroidia					32	4e-01	2e-01	12	4e-01	2e-01	29	4e-01	2e-01	0.637
Bacteroidetes	Bacteroidia	Bacteroidales				32	4e-01	2e-01	12	4e-01	2e-01	29	4e-01	2e-01	0.560
Bacteroidetes	Bacteroidia	Bacteroidales	Bacteroidaceae			32	3e-01	1e-01	12	2e-01	2e-01	29	3e-01	2e-01	0.493
Bacteroidetes	Bacteroidia	Bacteroidales	Bacteroidaceae	Bacteroidaceae_NI		32	3e-02	5e-02	11	1e-02	8e-03	29	1e-02	2e-02	**0.038**
Bacteroidetes	Bacteroidia	Bacteroidales	Bacteroidaceae	Bacteroidaceae_NI	Bacteroidaceae_NI_NI	32	3e-02	5e-02	11	1e-02	8e-03	29	1e-02	2e-02	**0.037**
Bacteroidetes	Bacteroidia	Bacteroidales	Bacteroidaceae	Bacteroides		32	2e-01	1e-01	12	2e-01	1e-01	29	2e-01	2e-01	0.703
Bacteroidetes	Bacteroidia	Bacteroidales	Bacteroidaceae	Bacteroides	Bacteroides_NI	32	3e-02	2e-02	12	2e-02	2e-02	29	2e-02	3e-02	0.099
Bacteroidetes	Bacteroidia	Bacteroidales	Bacteroidaceae	Bacteroides	caccae	27	1e-02	2e-02	7	3e-03	5e-03	16	5e-03	8e-03	**0.011**
Bacteroidetes	Bacteroidia	Bacteroidales	Bacteroidaceae	Bacteroides	cellulosilyticus	20	2e-03	5e-03	4	2e-04	4e-04	14	1e-03	3e-03	0.072
Bacteroidetes	Bacteroidia	Bacteroidales	Bacteroidaceae	Bacteroides	clarus	13	5e-04	1e-03	5	1e-03	2e-03	13	3e-03	7e-03	0.614
Bacteroidetes	Bacteroidia	Bacteroidales	Bacteroidaceae	Bacteroides	dorei	29	3e-02	5e-02	10	2e-02	4e-02	23	1e-02	3e-02	**0.026**
Bacteroidetes	Bacteroidia	Bacteroidales	Bacteroidaceae	Bacteroides	eggerthii	22	1e-02	2e-02	6	4e-03	1e-02	18	8e-03	2e-02	0.255
Bacteroidetes	Bacteroidia	Bacteroidales	Bacteroidaceae	Bacteroides	faecichinchillae	11	4e-04	8e-04	6	2e-04	4e-04	9	5e-04	1e-03	0.603
Bacteroidetes	Bacteroidia	Bacteroidales	Bacteroidaceae	Bacteroides	finegoldii	12	1e-03	3e-03	5	4e-04	5e-04	13	2e-03	5e-03	0.987

[illegible]	[illegible]	[illegible]	[illegible]	[illegible]	[illegible]	[illegible]	[illegible]	[illegible]	[illegible]	[illegible]	[illegible]	[illegible]	[illegible]	[illegible]	[illegible]
Bacteroidetes	Bacteroidia	Bacteroidales	Bacteroidaceae	Bacteroides	fragilis	10	3e-03	1e-02	7	4e-03	1e-02	14	1e-02	5e-02	0.240
Bacteroidetes	Bacteroidia	Bacteroidales	Bacteroidaceae	Bacteroides	intestinalis	22	2e-03	3e-03	4	3e-04	4e-04	16	2e-03	3e-03	0.107
Bacteroidetes	Bacteroidia	Bacteroidales	Bacteroidaceae	Bacteroides	massiliensis	23	2e-02	3e-02	10	2e-02	3e-02	22	2e-02	3e-02	0.919
Bacteroidetes	Bacteroidia	Bacteroidales	Bacteroidaceae	Bacteroides	ovatus	27	3e-03	5e-03	12	2e-03	2e-03	23	4e-03	9e-03	0.746
Bacteroidetes	Bacteroidia	Bacteroidales	Bacteroidaceae	Bacteroides	plebeius	17	2e-02	3e-02	6	3e-02	1e-01	17	1e-02	3e-02	0.926
Bacteroidetes	Bacteroidia	Bacteroidales	Bacteroidaceae	Bacteroides	stercoris	16	3e-03	6e-03	8	6e-03	1e-02	17	1e-02	2e-02	0.499
Bacteroidetes	Bacteroidia	Bacteroidales	Bacteroidaceae	Bacteroides	thetaiotaomicron	31	3e-03	4e-03	12	3e-03	4e-03	27	3e-03	5e-03	0.869
Bacteroidetes	Bacteroidia	Bacteroidales	Bacteroidaceae	Bacteroides	uniformis	32	3e-02	4e-02	12	2e-02	1e-02	29	3e-02	4e-02	0.485
Bacteroidetes	Bacteroidia	Bacteroidales	Bacteroidaceae	Bacteroides	vulgatus	32	6e-02	6e-02	11	4e-02	4e-02	28	7e-02	7e-02	0.545
Bacteroidetes	Bacteroidia	Bacteroidales	Bacteroidaceae	Bacteroides	xylanisolvens	29	3e-03	4e-03	11	4e-03	7e-03	26	2e-03	3e-03	0.999
Bacteroidetes	Bacteroidia	Bacteroidales	Porphyromonadaceae			32	5e-02	3e-02	12	4e-02	3e-02	29	3e-02	2e-02	**0.023**
Bacteroidetes	Bacteroidia	Bacteroidales	Porphyromonadaceae	Barnesiella		24	8e-03	1e-02	9	5e-03	8e-03	19	6e-03	9e-03	0.516
Bacteroidetes	Bacteroidia	Bacteroidales	Porphyromonadaceae	Barnesiella	Barnesiella_NI	23	4e-03	7e-03	6	1e-03	3e-03	13	1e-03	3e-03	**0.040**
Bacteroidetes	Bacteroidia	Bacteroidales	Porphyromonadaceae	Barnesiella	intestinihominis	21	4e-03	6e-03	9	4e-03	6e-03	19	5e-03	8e-03	0.854
Bacteroidetes	Bacteroidia	Bacteroidales	Porphyromonadaceae	Butyricimonas		25	2e-03	3e-03	7	6e-04	9e-04	22	1e-03	2e-03	0.373
Bacteroidetes	Bacteroidia	Bacteroidales	Porphyromonadaceae	Butyricimonas	Butyricimonas_NI	19	6e-04	1e-03	7	3e-04	5e-04	14	3e-04	6e-04	0.362
Bacteroidetes	Bacteroidia	Bacteroidales	Porphyromonadaceae	Butyricimonas	virosa	16	7e-04	2e-03	2	1e-04	3e-04	13	6e-04	1e-03	0.201
Bacteroidetes	Bacteroidia	Bacteroidales	Porphyromonadaceae	Odoribacter		27	4e-03	4e-03	9	5e-03	7e-03	25	2e-03	2e-03	0.144
Bacteroidetes	Bacteroidia	Bacteroidales	Porphyromonadaceae	Odoribacter	Odoribacter_NI	22	7e-04	8e-04	5	7e-04	2e-03	20	3e-04	5e-04	0.257
Bacteroidetes	Bacteroidia	Bacteroidales	Porphyromonadaceae	Odoribacter	splanchnicus	27	3e-03	3e-03	9	4e-03	6e-03	24	1e-03	2e-03	0.081
Bacteroidetes	Bacteroidia	Bacteroidales	Porphyromonadaceae	Parabacteroides		32	2e-02	2e-02	12	1e-02	1e-02	28	1e-02	8e-03	0.175
Bacteroidetes	Bacteroidia	Bacteroidales	Porphyromonadaceae	Parabacteroides	distasonis	31	8e-03	9e-03	10	3e-03	2e-03	24	4e-03	5e-03	0.057
Bacteroidetes	Bacteroidia	Bacteroidales	Porphyromonadaceae	Parabacteroides	merdae	26	7e-03	9e-03	11	7e-03	8e-03	23	4e-03	6e-03	0.447
Bacteroidetes	Bacteroidia	Bacteroidales	Porphyromonadaceae	Parabacteroides	Parabacteroides_NI	24	1e-03	2e-03	9	2e-03	5e-03	24	5e-04	7e-04	0.490
Bacteroidetes	Bacteroidia	Bacteroidales	Porphyromonadaceae	Porphyromonadaceae_NI		32	2e-02	2e-02	12	1e-02	1e-02	28	9e-03	2e-02	0.152
Bacteroidetes	Bacteroidia	Bacteroidales	Porphyromonadaceae	Porphyromonadaceae_NI	Porphyromonadaceae_NI_NI	32	2e-02	2e-02	12	1e-02	1e-02	28	9e-03	2e-02	0.154
Bacteroidetes	Bacteroidia	Bacteroidales	Prevotellaceae			29	4e-02	6e-02	9	1e-01	2e-01	26	9e-02	1e-01	0.932
Bacteroidetes	Bacteroidia	Bacteroidales	Prevotellaceae	Paraprevotella		20	5e-03	7e-03	7	3e-03	4e-03	16	5e-03	1e-02	0.563
Bacteroidetes	Bacteroidia	Bacteroidales	Prevotellaceae	Paraprevotella	clara	17	2e-03	3e-03	5	1e-03	2e-03	12	1e-03	4e-03	0.302
Bacteroidetes	Bacteroidia	Bacteroidales	Prevotellaceae	Paraprevotella	Paraprevotella_NI	19	3e-03	4e-03	6	2e-03	3e-03	16	4e-03	8e-03	0.673
Bacteroidetes	Bacteroidia	Bacteroidales	Prevotellaceae	Prevotella		21	2e-02	5e-02	7	9e-02	2e-01	23	7e-02	1e-01	0.691
Bacteroidetes	Bacteroidia	Bacteroidales	Prevotellaceae	Prevotella	copri	19	2e-02	5e-02	6	7e-02	1e-01	20	6e-02	1e-01	0.723
Bacteroidetes	Bacteroidia	Bacteroidales	Prevotellaceae	Prevotella	Prevotella_NI	16	3e-03	5e-03	7	9e-03	2e-02	18	3e-03	6e-03	0.723
Bacteroidetes	Bacteroidia	Bacteroidales	Prevotellaceae	Prevotellaceae_NI		26	1e-02	2e-02	8	4e-02	6e-02	22	1e-02	3e-02	0.991
Bacteroidetes	Bacteroidia	Bacteroidales	Prevotellaceae	Prevotellaceae_NI	Prevotellaceae_NI_NI	26	1e-02	2e-02	8	4e-02	6e-02	22	1e-02	3e-02	0.892
Bacteroidetes	Bacteroidia	Bacteroidales	Rikenellaceae			31	8e-02	8e-02	12	2e-02	2e-02	29	3e-02	4e-02	**0.016**
Bacteroidetes	Bacteroidia	Bacteroidales	Rikenellaceae	Alistipes		31	6e-02	7e-02	11	2e-02	2e-02	29	3e-02	4e-02	**0.012**
Bacteroidetes	Bacteroidia	Bacteroidales	Rikenellaceae	Alistipes	Alistipes	21	2e-03	3e-03	6	2e-04	4e-04	15	9e-04	2e-03	0.062
Bacteroidetes	Bacteroidia	Bacteroidales	Rikenellaceae	Alistipes	Alistipes_NI	30	2e-02	3e-02	11	5e-03	7e-03	26	7e-03	1e-02	**0.006**

Bacteroidetes	Bacteroidia	Bacteroidales	Rikenellaceae	Alistipes	finegoldii	22	2e-03	5e-03	9	1e-03	1e-03	15	8e-04	2e-03	0.056
Bacteroidetes	Bacteroidia	Bacteroidales	Rikenellaceae	Alistipes	indistinctus	16	2e-03	5e-03	4	1e-03	4e-03	16	7e-04	2e-03	0.659
Bacteroidetes	Bacteroidia	Bacteroidales	Rikenellaceae	Alistipes	onderdonkii	26	1e-02	3e-02	8	3e-03	5e-03	27	9e-03	2e-02	0.413
Bacteroidetes	Bacteroidia	Bacteroidales	Rikenellaceae	Alistipes	putredinis	31	2e-02	2e-02	11	9e-03	1e-02	24	9e-03	1e-02	0.053
Bacteroidetes	Bacteroidia	Bacteroidales	Rikenellaceae	Alistipes	shahii	29	3e-03	3e-03	10	1e-03	1e-03	20	1e-03	2e-03	**0.011**
Bacteroidetes	Bacteroidia	Bacteroidales	Rikenellaceae	Rikenellaceae_NI		30	1e-02	2e-02	8	3e-03	7e-03	22	3e-03	6e-03	**0.037**
Bacteroidetes	Bacteroidia	Bacteroidales	Rikenellaceae	Rikenellaceae_NI	Rikenellaceae_NI_NI	30	1e-02	2e-02	8	3e-03	7e-03	22	3e-03	6e-03	0.050
Firmicutes						32	4e-01	1e-01	12	5e-01	2e-01	29	5e-01	2e-01	0.472
Firmicutes	Bacilli					32	1e-02	1e-02	12	1e-02	9e-03	29	2e-02	3e-02	0.106
Firmicutes	Bacilli	Bacillales				27	1e-03	1e-03	6	1e-04	2e-04	21	2e-03	5e-03	**0.003**
Firmicutes	Bacilli	Bacillales	Bacillaceae			23	7e-04	1e-03	4	9e-05	2e-04	19	2e-03	5e-03	**0.039**
Firmicutes	Bacilli	Bacillales	Bacillaceae	Bacillaceae_NI		23	7e-04	1e-03	4	9e-05	2e-04	19	9e-04	1e-03	**0.042**
Firmicutes	Bacilli	Bacillales	Bacillaceae	Bacillaceae_NI	Bacillaceae_NI_NI	23	7e-04	1e-03	4	9e-05	2e-04	19	9e-04	1e-03	**0.025**
Firmicutes	Bacilli	Lactobacillales				32	1e-02	1e-02	12	1e-02	9e-03	29	2e-02	3e-02	0.102
Firmicutes	Bacilli	Lactobacillales	Lactobacillaceae			29	5e-03	1e-02	12	9e-03	6e-03	28	8e-03	1e-02	**0.009**
Firmicutes	Bacilli	Lactobacillales	Lactobacillaceae	Lactobacillaceae_NI		15	1e-03	5e-03	5	4e-04	9e-04	14	1e-03	4e-03	0.855
Firmicutes	Bacilli	Lactobacillales	Lactobacillaceae	Lactobacillaceae_NI	Lactobacillaceae_NI_NI	15	1e-03	5e-03	5	4e-04	9e-04	14	1e-03	4e-03	0.815
Firmicutes	Bacilli	Lactobacillales	Lactobacillaceae	Lactobacillus		26	4e-03	8e-03	12	8e-03	6e-03	27	7e-03	1e-02	**0.004**
Firmicutes	Bacilli	Lactobacillales	Lactobacillaceae	Lactobacillus	Lactobacillus_NI	6	5e-05	1e-04	5	3e-04	8e-04	11	3e-04	1e-03	0.300
Firmicutes	Bacilli	Lactobacillales	Lactobacillaceae	Lactobacillus	rogosae	25	3e-03	8e-03	12	7e-03	6e-03	23	6e-03	1e-02	**0.024**
Firmicutes	Bacilli	Lactobacillales	Streptococcaceae			32	5e-03	8e-03	10	5e-03	6e-03	27	1e-02	2e-02	0.671
Firmicutes	Bacilli	Lactobacillales	Streptococcaceae	Streptococcus		29	5e-03	8e-03	10	5e-03	6e-03	25	1e-02	2e-02	0.538
Firmicutes	Bacilli	Lactobacillales	Streptococcaceae	Streptococcus	australis	13	1e-04	2e-04	5	2e-04	3e-04	12	2e-04	4e-04	0.752
Firmicutes	Bacilli	Lactobacillales	Streptococcaceae	Streptococcus	salivarius	24	1e-03	2e-03	9	1e-03	1e-03	21	1e-03	2e-03	0.585
Firmicutes	Bacilli	Lactobacillales	Streptococcaceae	Streptococcus	sinensis	11	1e-04	3e-04	6	2e-04	3e-04	13	3e-04	5e-04	0.446
Firmicutes	Bacilli	Lactobacillales	Streptococcaceae	Streptococcus	Streptococcus_NI	26	2e-03	4e-03	10	3e-03	4e-03	23	6e-03	1e-02	0.721
Firmicutes	Bacilli	Lactobacillales	Streptococcaceae	Streptococcus	thermophilus	20	1e-03	2e-03	7	6e-04	7e-04	22	5e-03	1e-02	0.091
Firmicutes	Clostridia					32	3e-01	1e-01	12	4e-01	2e-01	29	4e-01	1e-01	0.892
Firmicutes	Clostridia	Clostridiales				32	3e-01	1e-01	12	4e-01	2e-01	29	4e-01	1e-01	0.829
Firmicutes	Clostridia	Clostridiales	Catabacteriaceae			19	2e-04	2e-04	6	2e-04	3e-04	19	2e-04	3e-04	0.980
Firmicutes	Clostridia	Clostridiales	Catabacteriaceae	Catabacteriaceae_NI		19	2e-04	2e-04	6	2e-04	2e-04	19	2e-04	3e-04	0.997
Firmicutes	Clostridia	Clostridiales	Catabacteriaceae	Catabacteriaceae_NI	Catabacteriaceae_NI_NI	19	2e-04	2e-04	6	2e-04	2e-04	19	2e-04	3e-04	0.948
Firmicutes	Clostridia	Clostridiales	Christensenellaceae			30	2e-03	3e-03	9	3e-04	3e-04	25	2e-03	2e-03	0.121
Firmicutes	Clostridia	Clostridiales	Christensenellaceae	Christensenellaceae_NI		30	2e-03	3e-03	9	3e-04	3e-04	23	1e-03	2e-03	0.086
Firmicutes	Clostridia	Clostridiales	Christensenellaceae	Christensenellaceae_NI	Christensenellaceae_NI_NI	30	2e-03	3e-03	9	3e-04	3e-04	23	1e-03	2e-03	0.063
Firmicutes	Clostridia	Clostridiales	Clostridiaceae			32	4e-02	2e-02	12	5e-02	7e-02	29	5e-02	3e-02	0.081
Firmicutes	Clostridia	Clostridiales	Clostridiaceae	Clostridiaceae_NI		32	2e-02	1e-02	12	2e-02	1e-02	29	2e-02	2e-02	0.214
Firmicutes	Clostridia	Clostridiales	Clostridiaceae	Clostridiaceae_NI	Clostridiaceae_NI_NI	32	2e-02	1e-02	12	2e-02	1e-02	29	2e-02	2e-02	0.105
Firmicutes	Clostridia	Clostridiales	Clostridiaceae	Clostridium		32	2e-02	2e-02	12	3e-02	6e-02	29	3e-02	2e-02	**0.025**

Firmicutes	Clostridia	Clostridiales	Clostridiaceae	Clostridium	Clostridium_NI	32	1e-02	1e-02	12	2e-02	5e-02	29	2e-02	1e-02	0.272
Firmicutes	Clostridia	Clostridiales	Clostridiaceae	Clostridium	lactatifermentans	6	7e-04	3e-03	3	3e-03	5e-03	15	6e-03	1e-02	**0.008**
Firmicutes	Clostridia	Clostridiales	Clostridiales Family XIII. Incertae Sedis			14	2e-04	5e-04	5	2e-04	4e-04	19	3e-04	4e-04	0.250
Firmicutes	Clostridia	Clostridiales	Clostridiales Family XIII. Incertae Sedis	Clostridiales Family XIII. Incertae Sedis_NI		14	2e-04	4e-04	5	2e-04	4e-04	19	3e-04	4e-04	0.198
Firmicutes	Clostridia	Clostridiales	Clostridiales Family XIII. Incertae Sedis	Clostridiales Family XIII. Incertae Sedis_NI	Clostridiales Family XIII. Incertae Sedis_NI_NI	14	2e-04	4e-04	5	2e-04	4e-04	19	3e-04	4e-04	0.190
Firmicutes	Clostridia	Clostridiales	Eubacteriaceae			32	3e-02	3e-02	12	2e-02	1e-02	29	4e-02	3e-02	0.762
Firmicutes	Clostridia	Clostridiales	Eubacteriaceae	Eubacteriaceae_NI		32	1e-02	1e-02	12	8e-03	4e-03	29	1e-02	2e-02	0.800
Firmicutes	Clostridia	Clostridiales	Eubacteriaceae	Eubacteriaceae_NI	Eubacteriaceae_NI_NI	32	1e-02	1e-02	12	8e-03	4e-03	29	1e-02	2e-02	0.783
Firmicutes	Clostridia	Clostridiales	Eubacteriaceae	Eubacterium		32	2e-02	2e-02	12	1e-02	1e-02	29	2e-02	3e-02	0.687
Firmicutes	Clostridia	Clostridiales	Eubacteriaceae	Eubacterium	eligens	31	1e-02	3e-02	10	1e-02	2e-02	26	1e-02	2e-02	0.855
Firmicutes	Clostridia	Clostridiales	Eubacteriaceae	Eubacterium	Eubacterium_NI	21	1e-03	2e-03	11	1e-03	1e-03	26	2e-03	3e-03	0.110
Firmicutes	Clostridia	Clostridiales	Eubacteriaceae	Eubacterium	ramulus	18	4e-04	6e-04	5	1e-03	3e-03	18	7e-04	1e-03	0.293
Firmicutes	Clostridia	Clostridiales	Eubacteriaceae	Eubacterium	rectale	19	2e-04	3e-04	5	2e-04	4e-04	16	4e-04	8e-04	0.876
Firmicutes	Clostridia	Clostridiales	Eubacteriaceae	Eubacterium	siraeum	13	8e-04	2e-03	4	1e-03	4e-03	18	3e-03	1e-02	0.127
Firmicutes	Clostridia	Clostridiales	Eubacteriaceae	Eubacterium	ventriosum	18	1e-03	2e-03	6	5e-04	1e-03	14	8e-04	2e-03	0.831
Firmicutes	Clostridia	Clostridiales	Gracilibacteraceae			24	6e-03	2e-02	3	2e-04	5e-04	9	9e-04	3e-03	**0.002**
Firmicutes	Clostridia	Clostridiales	Gracilibacteraceae	Gracilibacteraceae_NI		24	6e-03	2e-02	3	2e-04	5e-04	9	9e-04	3e-03	**0.002**
Firmicutes	Clostridia	Clostridiales	Gracilibacteraceae	Gracilibacteraceae_NI	Gracilibacteraceae_NI_NI	24	6e-03	2e-02	3	2e-04	5e-04	9	9e-04	3e-03	**0.002**
Firmicutes	Clostridia	Clostridiales	Lachnospiraceae			32	1e-01	8e-02	12	2e-01	2e-01	29	1e-01	7e-02	0.389
Firmicutes	Clostridia	Clostridiales	Lachnospiraceae	[Ruminococcus]		32	6e-03	8e-03	12	1e-02	2e-02	29	7e-03	7e-03	0.095
Firmicutes	Clostridia	Clostridiales	Lachnospiraceae	[Ruminococcus]	[Ruminococcus]_NI	22	1e-03	2e-03	11	3e-03	4e-03	24	2e-03	3e-03	**0.042**
Firmicutes	Clostridia	Clostridiales	Lachnospiraceae	[Ruminococcus]	gnavus	29	4e-03	7e-03	12	1e-02	1e-02	29	5e-03	5e-03	**0.040**
Firmicutes	Clostridia	Clostridiales	Lachnospiraceae	Blautia		32	5e-03	1e-02	12	7e-03	1e-02	29	7e-03	8e-03	0.118
Firmicutes	Clostridia	Clostridiales	Lachnospiraceae	Blautia	Blautia_NI	30	3e-03	6e-03	12	4e-03	9e-03	29	3e-03	4e-03	0.077
Firmicutes	Clostridia	Clostridiales	Lachnospiraceae	Blautia	luti	15	3e-04	8e-04	6	8e-04	2e-03	19	9e-04	2e-03	0.327
Firmicutes	Clostridia	Clostridiales	Lachnospiraceae	Blautia	obeum	11	1e-04	2e-04	5	4e-05	7e-05	11	1e-04	3e-04	0.922
Firmicutes	Clostridia	Clostridiales	Lachnospiraceae	Blautia	producta	22	8e-04	2e-03	10	1e-03	2e-03	28	1e-03	1e-03	**0.011**
Firmicutes	Clostridia	Clostridiales	Lachnospiraceae	Blautia	wexlerae	23	1e-03	4e-03	8	6e-04	1e-03	20	7e-04	1e-03	0.706
Firmicutes	Clostridia	Clostridiales	Lachnospiraceae	Coprococcus		29	8e-03	2e-02	10	4e-03	5e-03	25	5e-03	2e-02	0.585
Firmicutes	Clostridia	Clostridiales	Lachnospiraceae	Coprococcus	comes	24	5e-03	2e-02	8	2e-03	4e-03	23	4e-03	2e-02	0.696
Firmicutes	Clostridia	Clostridiales	Lachnospiraceae	Coprococcus	eutactus	14	2e-03	4e-03	6	2e-03	3e-03	8	4e-04	1e-03	0.154
Firmicutes	Clostridia	Clostridiales	Lachnospiraceae	Dorea		27	5e-03	1e-02	11	7e-03	9e-03	28	7e-03	8e-03	0.117
Firmicutes	Clostridia	Clostridiales	Lachnospiraceae	Dorea	formicigenerans	18	4e-04	9e-04	7	5e-04	9e-04	21	8e-04	1e-03	0.139
Firmicutes	Clostridia	Clostridiales	Lachnospiraceae	Dorea	longicatena	26	5e-03	1e-02	11	6e-03	8e-03	28	6e-03	8e-03	0.142
Firmicutes	Clostridia	Clostridiales	Lachnospiraceae	Eubacterium		16	3e-03	9e-03	6	1e-02	4e-02	20	1e-03	3e-03	0.711
Firmicutes	Clostridia	Clostridiales	Lachnospiraceae	Eubacterium	hadrum	16	3e-03	9e-03	6	1e-02	4e-02	20	1e-03	3e-03	0.712
Firmicutes	Clostridia	Clostridiales	Lachnospiraceae	Lachnoclostridium		25	2e-03	3e-03	7	9e-04	1e-03	27	4e-03	9e-03	0.416
Firmicutes	Clostridia	Clostridiales	Lachnospiraceae	Lachnoclostridium	clostridioforme	23	1e-03	2e-03	7	8e-04	1e-03	22	3e-03	8e-03	0.531

Firmicutes	Clostridia	Clostridiales	Lachnospiraceae	Lachnoclostridium	Lachnoclostridium_NI	17	6e-04	2e-03	3	1e-04	3e-04	20	5e-04	2e-03	0.306
Firmicutes	Clostridia	Clostridiales	Lachnospiraceae	Lachnospiraceae_NI		32	6e-02	4e-02	12	5e-02	4e-02	29	6e-02	3e-02	0.446
Firmicutes	Clostridia	Clostridiales	Lachnospiraceae	Lachnospiraceae_NI	Lachnospiraceae_NI_NI	32	6e-02	4e-02	12	5e-02	4e-02	29	6e-02	3e-02	0.726
Firmicutes	Clostridia	Clostridiales	Lachnospiraceae	Roseburia		32	2e-02	3e-02	12	7e-02	8e-02	29	3e-02	3e-02	**0.006**
Firmicutes	Clostridia	Clostridiales	Lachnospiraceae	Roseburia	faecis	31	1e-02	1e-02	12	3e-02	5e-02	29	1e-02	1e-02	0.207
Firmicutes	Clostridia	Clostridiales	Lachnospiraceae	Roseburia	hominis	23	6e-04	1e-03	8	2e-03	3e-03	16	1e-03	3e-03	0.493
Firmicutes	Clostridia	Clostridiales	Lachnospiraceae	Roseburia	intestinalis	14	3e-04	6e-04	7	3e-03	7e-03	17	1e-03	3e-03	0.129
Firmicutes	Clostridia	Clostridiales	Lachnospiraceae	Roseburia	inulinivorans	27	2e-03	2e-03	11	1e-02	2e-02	24	5e-03	7e-03	0.056
Firmicutes	Clostridia	Clostridiales	Lachnospiraceae	Roseburia	Roseburia_NI	32	8e-03	2e-02	12	2e-02	2e-02	28	1e-02	2e-02	**0.007**
Firmicutes	Clostridia	Clostridiales	Lachnospiraceae	Ruminococcus		21	2e-03	4e-03	9	4e-03	6e-03	16	2e-03	3e-03	0.487
Firmicutes	Clostridia	Clostridiales	Lachnospiraceae	Ruminococcus	torques	19	2e-03	4e-03	8	2e-03	3e-03	16	1e-03	3e-03	0.684
Firmicutes	Clostridia	Clostridiales	Oscillospiraceae			30	2e-02	1e-02	11	9e-03	9e-03	28	1e-02	1e-02	0.115
Firmicutes	Clostridia	Clostridiales	Oscillospiraceae	Oscillibacter		27	2e-03	2e-03	11	1e-03	8e-04	23	1e-03	1e-03	0.074
Firmicutes	Clostridia	Clostridiales	Oscillospiraceae	Oscillibacter	Oscillibacter_NI	26	2e-03	2e-03	11	6e-04	6e-04	20	6e-04	7e-04	**0.048**
Firmicutes	Clostridia	Clostridiales	Oscillospiraceae	Oscillospiraceae_NI		29	1e-02	1e-02	10	8e-03	9e-03	28	9e-03	1e-02	0.144
Firmicutes	Clostridia	Clostridiales	Oscillospiraceae	Oscillospiraceae_NI	Oscillospiraceae_NI_NI	29	1e-02	1e-02	10	8e-03	9e-03	28	9e-03	1e-02	0.125
Firmicutes	Clostridia	Clostridiales	Peptococcaceae			26	5e-03	8e-03	7	3e-04	3e-04	21	2e-03	3e-03	**0.002**
Firmicutes	Clostridia	Clostridiales	Peptococcaceae	Peptococcaceae_NI		26	5e-03	8e-03	7	3e-04	3e-04	21	2e-03	3e-03	**0.002**
Firmicutes	Clostridia	Clostridiales	Peptococcaceae	Peptococcaceae_NI	Peptococcaceae_NI_NI	26	5e-03	8e-03	7	3e-04	3e-04	21	2e-03	3e-03	**0.002**
Firmicutes	Clostridia	Clostridiales	Peptostreptococcaceae			23	1e-03	3e-03	7	1e-03	2e-03	23	2e-03	3e-03	0.400
Firmicutes	Clostridia	Clostridiales	Peptostreptococcaceae	Peptostreptococcaceae_NI		23	1e-03	3e-03	7	1e-03	2e-03	23	2e-03	3e-03	0.397
Firmicutes	Clostridia	Clostridiales	Peptostreptococcaceae	Peptostreptococcaceae_NI	Peptostreptococcaceae_NI_NI	23	1e-03	3e-03	7	1e-03	2e-03	23	2e-03	3e-03	0.353
Firmicutes	Clostridia	Clostridiales	Ruminococcaceae			32	1e-01	7e-02	12	9e-02	4e-02	29	1e-01	6e-02	0.649
Firmicutes	Clostridia	Clostridiales	Ruminococcaceae	Faecalibacterium		32	7e-02	5e-02	12	5e-02	2e-02	29	5e-02	3e-02	0.074
Firmicutes	Clostridia	Clostridiales	Ruminococcaceae	Faecalibacterium	Faecalibacterium_NI	32	1e-02	7e-03	12	7e-03	5e-03	29	6e-03	4e-03	**0.026**
Firmicutes	Clostridia	Clostridiales	Ruminococcaceae	Faecalibacterium	prausnitzii	32	6e-02	4e-02	12	4e-02	2e-02	29	4e-02	3e-02	0.166
Firmicutes	Clostridia	Clostridiales	Ruminococcaceae	Ruminiclostridium		14	6e-04	1e-03	6	6e-04	2e-03	17	3e-03	8e-03	0.257
Firmicutes	Clostridia	Clostridiales	Ruminococcaceae	Ruminiclostridium	Ruminiclostridium_NI	6	8e-05	4e-04	4	1e-04	3e-04	15	2e-03	6e-03	**0.007**
Firmicutes	Clostridia	Clostridiales	Ruminococcaceae	Ruminococcaceae_NI		32	4e-02	3e-02	12	3e-02	2e-02	29	5e-02	3e-02	0.529
Firmicutes	Clostridia	Clostridiales	Ruminococcaceae	Ruminococcaceae_NI	Ruminococcaceae_NI_NI	32	4e-02	3e-02	12	3e-02	2e-02	29	5e-02	3e-02	0.374
Firmicutes	Clostridia	Clostridiales	Ruminococcaceae	Ruminococcus		32	9e-03	1e-02	12	1e-02	1e-02	29	2e-02	2e-02	0.648
Firmicutes	Clostridia	Clostridiales	Ruminococcaceae	Ruminococcus	bromii	24	4e-03	5e-03	8	4e-03	5e-03	23	2e-03	5e-03	0.529
Firmicutes	Clostridia	Clostridiales	Ruminococcaceae	Ruminococcus	callidus	12	4e-04	1e-03	7	7e-04	9e-04	12	8e-04	2e-03	0.366
Firmicutes	Clostridia	Clostridiales	Ruminococcaceae	Ruminococcus	faecis	25	1e-03	2e-03	6	4e-03	9e-03	23	8e-03	2e-02	0.536
Firmicutes	Clostridia	Clostridiales	Ruminococcaceae	Ruminococcus	flavefaciens	10	3e-04	6e-04	5	4e-04	6e-04	6	2e-04	4e-04	0.389
Firmicutes	Clostridia	Clostridiales	Ruminococcaceae	Ruminococcus	gauvreauii	27	1e-03	1e-03	10	1e-03	2e-03	23	7e-04	9e-04	0.541
Firmicutes	Clostridia	Clostridiales	Ruminococcaceae	Ruminococcus	lactaris	15	9e-04	2e-03	5	1e-03	3e-03	9	3e-04	8e-04	0.316
Firmicutes	Clostridia	Clostridiales	Ruminococcaceae	Ruminococcus	Ruminococcus_NI	25	2e-03	3e-03	10	2e-03	6e-03	24	2e-03	3e-03	0.588
Firmicutes	Clostridia	Clostridiales	Ruminococcaceae	Subdoligranulum		29	2e-03	2e-03	10	3e-03	3e-03	28	4e-03	5e-03	0.076

Firmicutes	Clostridia	Clostridiales	Ruminococcaceae	Subdoligranulum	variabile	15	2e-04	4e-04	6	7e-04	1e-03	18	8e-04	1e-03	0.347
Firmicutes	Clostridia	Clostridiales	unclassified Clostridiales			31	4e-03	5e-03	12	2e-03	2e-03	26	4e-03	7e-03	0.946
Firmicutes	Clostridia	Clostridiales	unclassified Clostridiales	Flavonifractor		25	3e-03	5e-03	12	2e-03	2e-03	25	3e-03	6e-03	0.304
Firmicutes	Clostridia	Clostridiales	unclassified Clostridiales	Flavonifractor	Flavonifractor_NI	15	3e-04	5e-04	7	2e-04	3e-04	18	2e-04	3e-04	0.481
Firmicutes	Clostridia	Clostridiales	unclassified Clostridiales	Flavonifractor	plautii	22	3e-03	5e-03	11	2e-03	2e-03	23	3e-03	6e-03	0.382
Firmicutes	Clostridia	Clostridiales	unclassified Clostridiales	Pseudoflavonifractor		20	3e-04	3e-04	5	5e-05	8e-05	10	5e-04	1e-03	0.100
Firmicutes	Clostridia	Clostridiales	unclassified Clostridiales	Pseudoflavonifractor	Pseudoflavonifractor_NI	18	2e-04	3e-04	4	4e-05	8e-05	9	4e-04	1e-03	0.086
Firmicutes	Clostridia	Clostridiales	unclassified Clostridiales	unclassified Clostridiales_NI		24	4e-04	5e-04	9	3e-04	5e-04	20	4e-04	5e-04	0.994
Firmicutes	Clostridia	Clostridiales	unclassified Clostridiales	unclassified Clostridiales_NI	unclassified Clostridiales_NI_NI	24	4e-04	5e-04	9	3e-04	5e-04	20	4e-04	5e-04	0.972
Firmicutes	Clostridia	Clostridiales	Veillonellaceae			4	3e-05	1e-04	8	1e-03	2e-03	8	3e-04	1e-03	**0.042**
Firmicutes	Erysipelotrichi					25	4e-03	5e-03	7	2e-03	3e-03	22	6e-03	8e-03	0.664
Firmicutes	Erysipelotrichi	Erysipelotrichales				25	4e-03	5e-03	7	2e-03	3e-03	22	6e-03	8e-03	0.455
Firmicutes	Erysipelotrichi	Erysipelotrichales	Erysipelotrichaceae			25	4e-03	5e-03	7	2e-03	3e-03	22	6e-03	8e-03	0.455
Firmicutes	Erysipelotrichi	Erysipelotrichales	Erysipelotrichaceae	[Eubacterium]		10	3e-04	9e-04	7	1e-03	2e-03	18	3e-03	4e-03	**0.011**
Firmicutes	Erysipelotrichi	Erysipelotrichales	Erysipelotrichaceae	[Eubacterium]	biforme	6	3e-04	9e-04	5	1e-03	2e-03	14	2e-03	4e-03	**0.015**
Firmicutes	Erysipelotrichi	Erysipelotrichales	Erysipelotrichaceae	Erysipelotrichaceae_NI		22	4e-03	5e-03	7	6e-04	1e-03	14	3e-03	6e-03	0.119
Firmicutes	Erysipelotrichi	Erysipelotrichales	Erysipelotrichaceae	Erysipelotrichaceae_NI	Erysipelotrichaceae_NI_NI	22	4e-03	5e-03	7	6e-04	1e-03	14	3e-03	6e-03	0.132
Firmicutes	Erysipelotrichia					31	3e-03	6e-03	10	4e-03	4e-03	28	6e-03	9e-03	0.269
Firmicutes	Erysipelotrichia	Erysipelotrichales				31	3e-03	6e-03	10	4e-03	4e-03	28	6e-03	9e-03	0.248
Firmicutes	Erysipelotrichia	Erysipelotrichales	Erysipelotrichaceae			31	3e-03	6e-03	10	4e-03	4e-03	28	6e-03	9e-03	0.248
Firmicutes	Erysipelotrichia	Erysipelotrichales	Erysipelotrichaceae	Erysipelotrichaceae_NI		19	1e-03	5e-03	3	4e-05	1e-04	12	5e-04	1e-03	**0.020**
Firmicutes	Erysipelotrichia	Erysipelotrichales	Erysipelotrichaceae	Erysipelotrichaceae_NI	Erysipelotrichaceae_NI_NI	19	1e-03	5e-03	3	4e-05	1e-04	12	5e-04	1e-03	**0.014**
Firmicutes	Erysipelotrichia	Erysipelotrichales	Erysipelotrichaceae	Holdemania		20	3e-04	5e-04	7	1e-03	3e-03	17	6e-04	8e-04	0.758
Firmicutes	Erysipelotrichia	Erysipelotrichales	Erysipelotrichaceae	Holdemania	filiformis	20	3e-04	4e-04	6	1e-03	3e-03	17	5e-04	8e-04	0.801
Firmicutes	Negativicutes					32	7e-02	1e-01	12	1e-01	1e-01	29	9e-02	1e-01	0.953
Firmicutes	Negativicutes	Selenomonadales				32	7e-02	1e-01	12	1e-01	1e-01	29	9e-02	1e-01	0.366
Firmicutes	Negativicutes	Selenomonadales	Acidaminococcaceae			27	1e-02	2e-02	10	3e-02	4e-02	29	2e-02	3e-02	0.437
Firmicutes	Negativicutes	Selenomonadales	Acidaminococcaceae	Phascolarctobacterium		27	1e-02	2e-02	9	2e-02	4e-02	27	2e-02	3e-02	0.483
Firmicutes	Negativicutes	Selenomonadales	Acidaminococcaceae	Phascolarctobacterium	faecium	16	8e-03	2e-02	7	1e-02	2e-02	18	1e-02	2e-02	0.476
Firmicutes	Negativicutes	Selenomonadales	Acidaminococcaceae	Phascolarctobacterium	Phascolarctobacterium_NI	18	1e-03	2e-03	5	5e-03	1e-02	18	3e-03	9e-03	0.492
Firmicutes	Negativicutes	Selenomonadales	Veillonellaceae			30	6e-02	1e-01	11	9e-02	1e-01	25	6e-02	1e-01	0.280
Firmicutes	Negativicutes	Selenomonadales	Veillonellaceae	Allisonella		8	2e-04	6e-04	6	8e-04	1e-03	10	6e-04	1e-03	0.182
Firmicutes	Negativicutes	Selenomonadales	Veillonellaceae	Allisonella	histaminiformans	6	1e-04	3e-04	6	6e-04	1e-03	10	4e-04	8e-04	0.093
Firmicutes	Negativicutes	Selenomonadales	Veillonellaceae	Dialister		21	4e-02	1e-01	7	5e-02	7e-02	15	2e-02	7e-02	0.395

Firmicutes	Negativicutes	Selenomonadales	Veillonellaceae	Dialister	Dialister_NI	16	1e-02	2e-02	7	2e-02	5e-02	11	1e-02	3e-02	0.460
Firmicutes	Negativicutes	Selenomonadales	Veillonellaceae	Veillonella		7	4e-04	1e-03	6	3e-03	9e-03	10	6e-04	2e-03	0.373
Firmicutes	Negativicutes	Selenomonadales	Veillonellaceae	Veillonella	Veillonella_NI	6	3e-04	1e-03	6	2e-03	7e-03	7	2e-04	8e-04	0.087
Firmicutes	Negativicutes	Selenomonadales	Veillonellaceae	Veillonellaceae_NI		23	1e-02	2e-02	5	1e-02	4e-02	17	2e-02	3e-02	0.508
Firmicutes	Negativicutes	Selenomonadales	Veillonellaceae	Veillonellaceae_NI	Veillonellaceae_NI_NI	23	1e-02	2e-02	5	1e-02	4e-02	17	2e-02	3e-02	0.390
Proteobacteria						32	1e-01	8e-02	12	7e-02	4e-02	29	1e-01	1e-01	0.474
Proteobacteria	Alphaproteobacteria					32	3e-02	5e-02	12	3e-02	3e-02	29	6e-02	7e-02	0.338
Proteobacteria	Alphaproteobacteria	Rhizobiales				32	3e-02	5e-02	12	3e-02	3e-02	29	6e-02	7e-02	0.384
Proteobacteria	Alphaproteobacteria	Rhizobiales	Hyphomicrobiaceae			32	3e-02	5e-02	12	3e-02	3e-02	29	6e-02	7e-02	0.385
Proteobacteria	Alphaproteobacteria	Rhizobiales	Hyphomicrobiaceae	Gemmiger		32	3e-02	5e-02	12	3e-02	3e-02	29	6e-02	7e-02	0.307
Proteobacteria	Alphaproteobacteria	Rhizobiales	Hyphomicrobiaceae	Gemmiger	formicilis	32	3e-02	5e-02	12	3e-02	3e-02	29	6e-02	7e-02	0.363
Proteobacteria	Betaproteobacteria					30	1e-02	2e-02	11	1e-02	1e-02	26	1e-02	2e-02	0.268
Proteobacteria	Betaproteobacteria	Burkholderiales				30	1e-02	2e-02	11	1e-02	1e-02	26	9e-03	2e-02	0.684
Proteobacteria	Betaproteobacteria	Burkholderiales	Oxalobacteraceae			18	2e-03	4e-03	5	1e-03	2e-03	14	1e-03	3e-03	0.599
Proteobacteria	Betaproteobacteria	Burkholderiales	Sutterellaceae			29	1e-02	2e-02	11	1e-02	9e-03	26	8e-03	1e-02	0.867
Proteobacteria	Betaproteobacteria	Burkholderiales	Sutterellaceae	Parasutterella		19	5e-03	1e-02	6	3e-03	5e-03	13	3e-03	7e-03	0.599
Proteobacteria	Betaproteobacteria	Burkholderiales	Sutterellaceae	Parasutterella	excrementihominis	18	4e-03	1e-02	6	2e-03	4e-03	13	2e-03	5e-03	0.631
Proteobacteria	Betaproteobacteria	Burkholderiales	Sutterellaceae	Sutterella		17	2e-03	4e-03	10	5e-03	6e-03	14	2e-03	5e-03	0.095
Proteobacteria	Betaproteobacteria	Burkholderiales	Sutterellaceae	Sutterella	Sutterella_NI	10	3e-04	9e-04	5	6e-04	2e-03	8	7e-04	2e-03	0.983
Proteobacteria	Deltaproteobacteria					26	6e-03	8e-03	8	4e-03	8e-03	25	4e-03	5e-03	0.074
Proteobacteria	Deltaproteobacteria	Desulfovibrionales				24	6e-03	8e-03	8	4e-03	8e-03	25	4e-03	5e-03	0.434
Proteobacteria	Deltaproteobacteria	Desulfovibrionales	Desulfovibrionaceae			24	6e-03	8e-03	8	4e-03	8e-03	25	4e-03	5e-03	0.434
Proteobacteria	Deltaproteobacteria	Desulfovibrionales	Desulfovibrionaceae	Bilophila		18	2e-03	3e-03	6	6e-04	9e-04	16	1e-03	3e-03	0.511
Proteobacteria	Deltaproteobacteria	Desulfovibrionales	Desulfovibrionaceae	Bilophila	Bilophila_NI	14	5e-04	1e-03	5	3e-04	6e-04	12	3e-04	6e-04	0.891
Proteobacteria	Deltaproteobacteria	Desulfovibrionales	Desulfovibrionaceae	Bilophila	wadsworthia	18	1e-03	2e-03	5	3e-04	6e-04	15	1e-03	2e-03	0.371
Proteobacteria	Deltaproteobacteria	Desulfovibrionales	Desulfovibrionaceae	Desulfovibrio		15	4e-03	6e-03	4	3e-03	8e-03	18	3e-03	4e-03	0.528
Proteobacteria	Deltaproteobacteria	Desulfovibrionales	Desulfovibrionaceae	Desulfovibrio	Desulfovibrio_NI	10	2e-03	4e-03	3	2e-03	8e-03	15	9e-04	2e-03	0.616
Proteobacteria	Gammaproteobacteria					31	4e-02	7e-02	11	2e-02	3e-02	28	4e-02	9e-02	0.106
Proteobacteria	Gammaproteobacteria	Enterobacteriales				30	4e-02	7e-02	10	2e-02	2e-02	27	4e-02	8e-02	0.326
Proteobacteria	Gammaproteobacteria	Enterobacteriales	Enterobacteriaceae			30	4e-02	7e-02	10	2e-02	2e-02	27	4e-02	8e-02	0.326
Proteobacteria	Gammaproteobacteria	Enterobacteriales	Enterobacteriaceae	Enterobacteriaceae_NI		30	4e-02	7e-02	10	2e-02	2e-02	27	3e-02	8e-02	0.328
Proteobacteria	Gammaproteobacteria	Enterobacteriales	Enterobacteriaceae	Enterobacteriaceae_NI	Enterobacteriaceae_NI_NI	30	4e-02	7e-02	10	2e-02	2e-02	27	3e-02	8e-02	0.448
Synergistetes						19	3e-03	7e-03	5	2e-04	3e-04	12	6e-04	2e-03	0.066
Synergistetes	Synergistia					19	3e-03	7e-03	5	2e-04	3e-04	12	6e-04	2e-03	0.064
Synergistetes	Synergistia	Synergistales				19	3e-03	7e-03	5	2e-04	3e-04	12	6e-04	2e-03	0.066
Synergistetes	Synergistia	Synergistales	Synergistaceae			19	3e-03	7e-03	5	2e-04	3e-04	12	6e-04	2e-03	0.066
Synergistetes	Synergistia	Synergistales	Synergistaceae	Synergistaceae_NI		19	2e-03	6e-03	4	2e-04	3e-04	11	6e-04	2e-03	**0.031**
Synergistetes	Synergistia	Synergistales	Synergistaceae	Synergistaceae_NI	Synergistaceae_NI_NI	19	2e-03	6e-03	4	2e-04	3e-04	11	6e-04	2e-03	**0.042**
Tenericutes						17	5e-03	8e-03	3	7e-04	2e-03	6	1e-03	3e-03	**0.007**

Tenericutes	Mollicutes					17	5e-03	8e-03	3	7e-04	2e-03	6	1e-03	3e-03	**0.005**
Verrucomicrobia						20	2e-03	4e-03	3	4e-04	7e-04	13	7e-04	1e-03	**0.029**
Verrucomicrobia	Verrucomicrobiae					18	2e-03	3e-03	3	4e-04	7e-04	13	6e-04	1e-03	0.431
Verrucomicrobia	Verrucomicrobiae	Verrucomicrobiales				18	2e-03	3e-03	3	4e-04	7e-04	13	6e-04	1e-03	0.100
Verrucomicrobia	Verrucomicrobiae	Verrucomicrobiales	Verrucomicrobiaceae			18	2e-03	3e-03	3	4e-04	7e-04	13	6e-04	1e-03	0.100
Verrucomicrobia	Verrucomicrobiae	Verrucomicrobiales	Verrucomicrobiaceae	Akkermansia		18	2e-03	3e-03	3	4e-04	7e-04	13	6e-04	1e-03	0.100
Verrucomicrobia	Verrucomicrobiae	Verrucomicrobiales	Verrucomicrobiaceae	Akkermansia	muciniphila	18	2e-03	3e-03	3	4e-04	7e-04	13	6e-04	1e-03	0.078

Supplementary data Table S2:

Family	Microbiotype 1	Microbiotype 2	Microbiotype 3	Microbiotype 4
Bacteroidaceae	0.257 (±0.102)	0.121 (±0.078)	0.098 (±0.090)	0.412 (±0.169)
Ruminococcaceae	0.238 (±0.075)	0.280 (±0.084)	0.193 (±0.118)	0.103 (±0.067)
Lachnospiraceae	0.221 (±0.090)	0.177 (±0.086)	0.362 (±0.179)	0.209 (±0.114)
Christensenellaceae	0.029 (±0.032)	0.041 (±0.037)	0.015 (±0.027)	0.004 (±0.010)
Tannerellaceae	0.028 (±0.024)	0.019 (±0.017)	0.009 (±0.016)	0.039 (±0.054)
Barnesiellaceae	0.022 (±0.022)	0.013 (±0.016)	0.004 (±0.009)	0.009 (±0.015)
Erysipelotrichaceae	0.018 (±0.021)	0.022 (±0.021)	0.036 (±0.048)	0.015 (±0.024)
Enterobacteriaceae	0.017 (±0.042)	0.027 (±0.065)	0.041 (±0.102)	0.041 (±0.103)
Bifidobacteriaceae	0.017 (±0.030)	0.015 (±0.022)	0.032 (±0.049)	0.015 (±0.033)
Rikenellaceae	0.017 (±0.017)	0.012 (±0.014)	0.006 (±0.011)	0.008 (±0.014)
Prevotellaceae	NC	0.036 (±0.079)	0.024 (±0.072)	0.034 (±0.100)
Akkermansiaceae	0.015 (±0.034)	0.030 (±0.043)	0.031 (±0.069)	NC
Veillonellaceae	0.010 (±0.019)	0.012 (±0.029)	0.021 (±0.043)	0.025 (±0.065)
Peptostreptococcaceae	0.007 (±0.010)	0.007 (±0.011)	0.013 (±0.025)	0.004 (±0.011)
Burkholderiaceae	0.007 (±0.009)	NC	NC	0.009 (±0.014)
Clostridiaceae_1	0.006 (±0.011)	0.009 (±0.015)	0.013 (±0.027)	0.003 (±0.007)
Desulfovibrionaceae	0.004 (±0.005)	0.005 (±0.005)	0.002 (±0.004)	0.003 (±0.007)
Marinifilaceae	0.004 (±0.004)	0.004 (±0.006)	0.002 (±0.005)	0.004 (±0.005)
Streptococcaceae	0.004 (±0.010)	NC	0.012 (±0.020)	0.005 (±0.013)
Family_XIII	0.003 (±0.003)	0.005 (±0.004)	0.004 (±0.005)	0.002 (±0.004)
Eggerthellaceae	NC	NC	0.003 (±0.005)	0.001 (±0.003)
